# Comparing Biomarkers for Predicting Pathological Responses to Neoadjuvant Therapy in HER2-Positive Breast Cancer: A Systematic Review and Meta-Analysis

**DOI:** 10.3389/fonc.2021.731148

**Published:** 2021-10-28

**Authors:** Fuxing Zhao, Xingfa Huo, Miaozhou Wang, Zhen Liu, Yi Zhao, Dengfeng Ren, Qiqi Xie, Zhilin Liu, Zitao Li, Feng Du, Guoshuang Shen, Jiuda Zhao

**Affiliations:** ^1^ Breast Disease Diagnosis and Treatment Center of Affiliated Hospital of Qinghai University & Affiliated Cancer Hospital of Qinghai University, Xining, China; ^2^ Key Laboratory of Carcinogenesis and Translational Research (Ministry of Education), The VIPII Gastrointestinal Cancer Division of Medical Department, Peking University Cancer Hospital and Institute, Beijing, China

**Keywords:** HER2-enriched subtype, breast cancer, biomarker, predict, diagnostic

## Abstract

**Introduction:**

The predictive strength and accuracy of some biomarkers for the pathological complete response (pCR) to neoadjuvant therapy for HER2-positive breast cancer remain unclear. This study aimed to compare the accuracy of the HER2-enriched subtype and the presence of PIK3CA mutations, namely, TILs, HRs, and Ki-67, in predicting the pCR to HER2-positive breast cancer therapy.

**Methods:**

We screened studies that included pCR predicted by one of the following biomarkers: the HER2-enriched subtype and the presence of PIK3CA mutations, TILs, HRs, or Ki-67. We then calculated the pooled sensitivity, specificity, positive and negative predictive values (PPVs and NPVs, respectively), and positive and negative likelihood ratios (LRs). Summary receiver operating characteristic (SROC) curves and areas under the curve (AUCs) were used to estimate the diagnostic accuracy.

**Results:**

The pooled estimates of sensitivity and specificity for the HER2-enriched subtype and the presence of PIK3CA mutations, namely, TILs, HRs, and Ki-67, were 0.66 and 0.62, 0.85 and 0.27, 0.49 and 0.61, 0.54 and 0.64, and 0.68 and 0.51, respectively. The AUC of the HER2-enriched subtype was significantly higher (0.71) than those for the presence of TILs (0.59, *p* = 0.003), HRs (0.65, *p* = 0.003), and Ki-67 (0.62, *p* = 0.005). The AUC of the HER2-enriched subtype had a tendency to be higher than that of the presence of PIK3CA mutations (0.58, *p* = 0.220). Moreover, it had relatively high PPV (0.58) and LR+ (1.77), similar NPV (0.73), and low LR− (0.54) compared with the other four biomarkers.

**Conclusions:**

The HER2-enriched subtype has a moderate breast cancer diagnostic accuracy, which is better than those of the presence of PIK3CA mutations, TILs, HRs, and Ki-67.

## Introduction

In approximately 20% of breast cancer cases, the expression of human epidermal growth factor receptor (HER2), which is associated with a poor prognosis, is enhanced (1–3). Neoadjuvant therapy can increase the operability rate for locally advanced diseases and inflammatory subtypes and increase the possibility of breast conservation by reducing tumor bulk or downstaging the tumor (4–6). HER2-targeted therapies such as treatments with trastuzumab, pertuzumab, lapatinib, and trastuzumab emtansine (T-DM1), neratinb, tucatinib, and trastuzumab–deruxtecan, have shown clinically significant efficacy against HER2-positive breast cancer. The National Comprehensive Cancer Network Guidelines, Version 7 (2021), recommend chemotherapy and trastuzumab-based therapy as preoperative systemic therapies for HER2-positive breast cancer. The guidelines also suggest that a pertuzumab-containing regimen is useful for patients with T2 or N1 HER2-positive, early-stage breast cancer in a neoadjuvant setting (7).

Single or dual HER2 blockades in combination with chemotherapy have achieved a pathologically complete response (pCR) of >60% for HER2-positive breast cancer (6, 8, 9). The achievement of pCR has significantly improved the long-term patient outcomes in HER2-positive breast cancer (6, 10–13). However, not all HER2-positive patients can achieve pCR when receiving HER2-targeted neoadjuvant therapy. Selecting patients who can achieve pCR based on biomarkers has thus become a vital clinical issue. To date, multiple potential biomarkers have been investigated among trials involving neoadjuvant therapies. In addition to HER2 overexpression or amplification, the most reported predictive biomarkers for patients with HER2-positive cancer include the HER2-enriched subtype and the presence of phosphatase phosphoinositol-3 (PI3) kinase (PIK3CA) mutations, tumor-infiltrating lymphocytes (TILs), hormone receptors (HRs), and Ki-67.

The HER2-enriched subtype is identified based on the PAM50 signature, which describes the expression profiles of 50 genes; intrinsic typing of PAM50 is now widely used in breast cancer research (14–16). High ERBB2 mRNA and protein levels appear to be associated with activation of the EGFR-HER2 signaling pathway (15–17). PIK3CA is present in the HER2 downstream signaling pathway, and the mutation of PIK3CA or the loss of PTEN can activate the PI3K pathway in breast cancer (18, 19). The PI3K pathway is associated with resistance to HER2-targeted therapy. Activation of PIK3CA mutations and deletion of PTEN (PTEN is a key negative regulator of PI3K signaling) lead to resistance to trastuzumab and lapatinib in breast cancer cell lines, and low PTEN levels are associated with worse patient prognosis (20–22). It was shown that the addition of additional targeted agents for PIK3CA mutations did not show additional benefit in terms of sensitivity to HER2-targeted therapy in the BOLERO-2/3 trial (22, 23). TIL is a stroma component that acts as an important mediator of tumor immunity. It has been shown that TIL is associated with improved distant-metastasis-free survival and increased (pCR) rates of neoadjuvant trastuzumab and chemotherapy in patients with HER2-positive early-stage breast cancer (20, 22). HR engages in crosstalk with HER2-receptor-mediated pathways (24, 25). Ki-67 is a marker of cell proliferation and is specifically expressed in the nucleus in G1 through M phases of the cell cycle (26). All these factors are directly or indirectly involved in the HER2 signaling pathway and may influence the effectiveness of HER2-targeted drugs. Multiple trials and meta-analyses have shown that these five factors can act as biomarkers for predicting pCR to neoadjuvant therapy with HER2-targeted drugs in patients with HER2-positive cancer (27–35).

Our study aimed to perform a systematic review to compare the relative diagnostic accuracy of HER2-enriched subtypes and the presence of PIK3CA mutations, namely, TILs, HRs, and Ki-67, in predicting the degree of pCR to neoadjuvant therapy with HER2-targeted drugs in patients with HER2-positive breast cancer.

## Methods

### Study Design

This meta-analysis was performed in accordance with the Preferred Reporting Items for Systematic Reviews and Meta-Analyses guideline (36). Two reviewers (FZ and XH) independently performed the literature search, assessed the eligibility criteria of related studies reported in the literature, and performed data extraction.

### Search Strategy

To identify clinical trials that assessed the potential of biomarkers in predicting pCR to HER2-targeted therapies, a systematic search was performed in the PubMed/MEDLINE and Embase databases, and reports were obtained from several main congresses of the European Society of Medical Oncology, the American Society of Clinical Oncology, and the San Antonio Breast Cancer Symposium databases. Reports included in this study were published between January 1, 2000 and September 30, 2020. The search strategy was based on the following combination of tags: (a) neoadjuvant OR preoperative, (b) breast cancer OR breast neoplasm OR breast carcinoma, (c) HER2-enriched subtype OR HER2-E, (d) phosphatase phosphoinositol-3 kinase mutation OR PIK3CA mutation, (e) tumor-infiltrating lymphocytes OR TIL, (f) hormone receptor OR estrogen receptor OR progesterone receptor, and (g) Ki-67 index OR Ki-67. The complete search information is presented in **Supplementary Table S1**.

### Eligibility Criteria

The inclusion criteria were as follows: (a) pathological results were reported after neoadjuvant therapy and surgery in stage I to stage III HER2-positive breast cancer; (b) patients who received HER2-targeted drugs that were used as part of the neoadjuvant therapy in prospective randomized or single-arm trials that specified the presence of a HER2-enriched subtype, PIK3CA mutation, TILs, HRs, or Ki-67 (retrospective studies were also included for Ki-67 because few prospective studies reported the results for Ki-67); (c) the presence of a HER2-enriched subtype, PIK3CA mutations, TILs, HRs, or Ki-67 was prospectively or retrospectively used to predict pCR in the abovementioned trials; and (d) articles were written in English. Different definitions of pCR in studies were allowed. Letters to the editor, reviews, editorials, comments, case reports, and studies involving ≤10 patients were excluded. For duplicate publications describing the same populations or overlapping patient cohorts, only the largest, most recent publication was included. Any discrepancies between reviewers were resolved through a discussion until a consensus was reached.

### Data Extraction and Quality Assessment

Two reviewers independently extracted patient characteristics and treatment and pathological information from all eligible studies. The primary study outcome was a comparison of the accuracy of the HER2-enriched subtype, PIK3CA mutations, TILs, HRs, and Ki-67 in predicting pCR rates when used as biomarkers. The following data were extracted from each study: study name, first author’s last name, study nation, publication year, study design characteristics, participant number, therapy regimens, HER2 status, biomarkers assessed, pCR definition, pCR rate, and, if possible, the cutoff value for biomarkers. Data describing true-positive (TP), false-positive (FP), true-negative (TN), and false-negative (FN) levels were extracted to construct 2 × 2 tables. If the study reported multiple biomarker tests, results describing pCR predictions based on individual biomarkers were extracted separately. The quality of the included studies was assessed using the Quality Assessment of Diagnostic Accuracy Studies (37–39).

### Statistical Analysis

We calculated the sensitivity, specificity, positive predictive value (PPV), negative predictive value (NPV), positive likelihood ratio (LR+), and negative LR (LR−) for data obtained from included studies, which were summarized in 2 × 2 tables containing TP, TN, FP, and FN values. Data were pooled together using the Moses–Littenberg model (fixed-effects model) and DerSimonian–Laird model (random-effects model) to generate unweighted and weighted linear regression models, respectively. We also developed summary receiver operating characteristic (SROC) curves and a Q* index. We additionally measured the relationship between test modalities and pCR using the SROC curves and the resultant relative area under the curve (AUC) values. Statistical comparisons of the AUCs were performed using the formula of Hanley and McNeil.

We calculated the pooled sensitivity and specificity for each modality and compared the overall differences in each modality. Random-effects models were used to address the anticipated heterogeneity. To estimate the publication bias for each study, we used the Stata 12.0 software to analyze all eligible studies using Deek’s test. All analyses were performed using the Meta-DiSc version 1.4 and Stata 12.0 software. All tests of statistical significance were two-sided, and a *p*-value of <0.05 was considered significant.

## Results

### Study Characteristics

**Figure 1:**
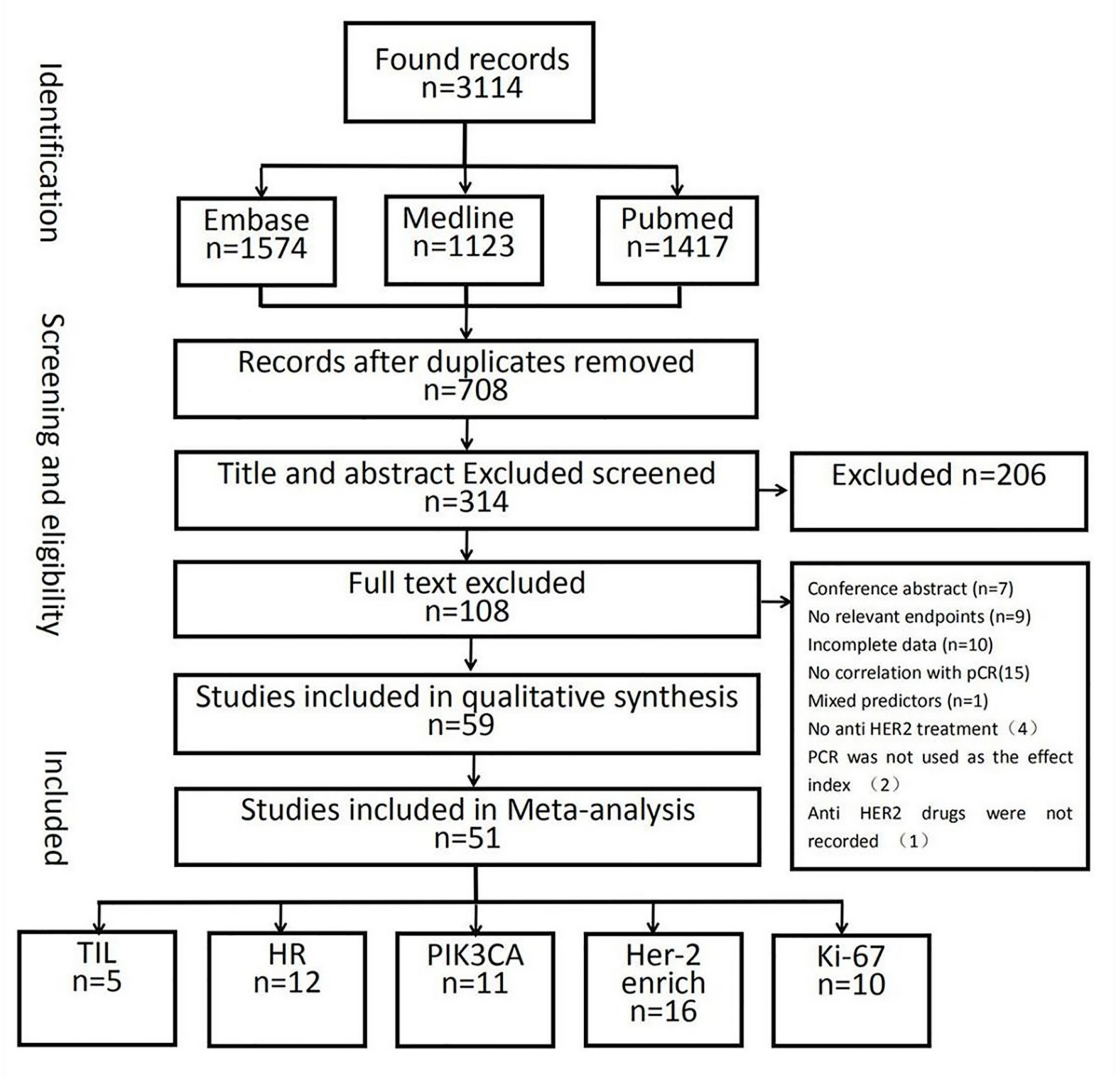
Flowchart illustrating the study-selection strategy.

**Figure 2:**
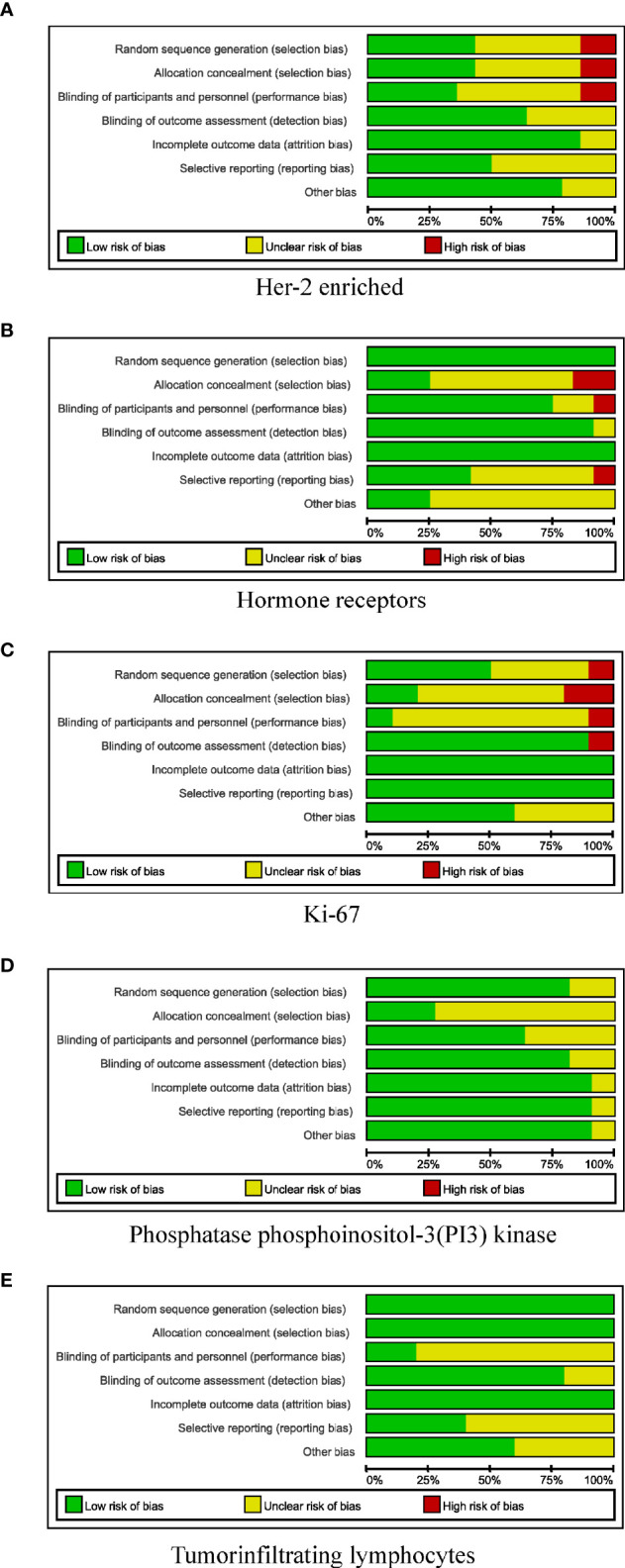
Risk-of-bias review of all included studies. **(A)** HER-2 enriched. **(B)** Hormone receptors. **(C)** Ki-67. **(D)** Phosphatase phosphoinositol-3 (PI3) kinase. **(E)** Tumor-infiltrating lymphocytes.

In total, 10,530 citations were identified. Of these studies, 51 (40–90) that met the inclusion criteria assessed the relationship between the pCR rate of non-adjuvant therapy with HER2-targeted drugs and either the HER2-enriched subtype (n = 16) (40–55) or the presence of HRs (n = 12) (55–66), Ki-67 (n = 10) (67–76), TILs (n = 5) (46, 77–80), or PIK3CA mutations (n = 11) (54, 81–90) (**Figure 1** and **Supplementary Table S2**). In total, 21 studies examined either multiple-patient cohorts or ≥2 individual predictive biomarkers, which resulted in a total of 94 individual analyses. Among all patients studied, 4,095 achieved pCR and 6,435 did not. **Supplementary Table S2** presents the primary features of these studies, including the study phase, study size, tumor stage, treatment, pCR rate, and pCR definition. Neoadjuvant therapies typically involved anthracycline–taxane combined with HER2-targeted drugs. The main HER2-targeted drugs were trastuzumab, pertuzumab, lapatinib, and T-DM1, which were used as single or dual HER2 blockades. The results of the quality assessment are presented in **Figure 2** (quality assessment results for each study are presented in **Supplementary Figure S1**).

### SROC Curves

**Supplementary Figure S2** shows a forest plot of the sensitivity and specificity of biomarkers in predicting pCR. The sensitivity of the HER2-enriched subtype and the presence of PIK3CA mutations, TILs, HRs, and Ki-67 ranged from 0.36 to 0.92, 0.71 to 0.92, 0.22 to 0.76, 0.20 to 0.83, and 0.00 to 1.00, respectively. Specificity values for the HER2-enriched subtype and the presence of PIK3CA mutations, TILs, HRs, and Ki-67 ranged from 0.33 to 0.88, 0.17 to 0.43, 0.18 to 0.96, 0.50 to 1.00, and 0.32 to 0.85, respectively.

**Figure 3:**
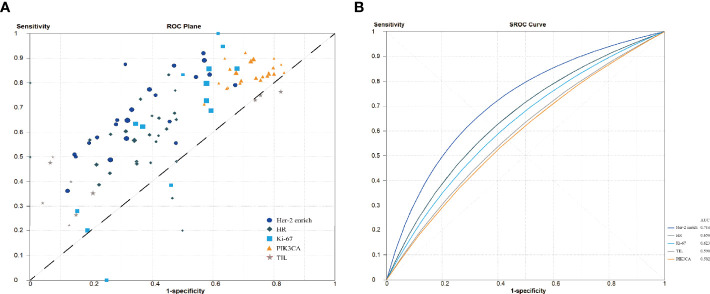
Summary receiver operating characteristic curve analysis. **(A)** Sensitivity and 1 − specificity values from each study. **(B)** Summary receiver operating characteristic curve. HR, hormone receptors; PIK3CA, phosphoinositide 3-kinases; TIL, tumor-infiltrating lymphocytes.

**Figure 3A** shows the derived sensitivity and 1 − specificity values for each study. **Figure 3B** shows the forest plot and SROC curves for sensitivity, specificity, and 95% CI for the HER2-enriched subtype and the presence of PIK3CA mutations, TILs, HRs, and Ki-67 of each study. The SROC curves were plotted based on weighting each study based on the number of samples. The weighted SROC curves suggested that the HER2-enriched subtype had better overall diagnostic accuracy; the AUC of the HER2-enriched subtype was significantly higher (0.71) than those of the presence of TILs (0.59, *p* = 0.003), HRs (0.65, *p* = 0.003), and Ki-67 (0.62, *p* =0.005); the presence of TILs had a relatively lower AUC at <0.60. The HER2-enriched subtype also showed a tendency to have better diagnostic accuracy with an AUC significantly higher (0.71) than that of the presence of PIK3CA mutations (0.58, *p* = 0.220; although the *p*-value is >0.05, it is clinically significant considering that its line is lower than that of the other four biomarkers and that it may be attribute to the wide 95% confidence interval and limited sample size, resulting in poor statistical efficiency; therefore, the difference between AUC values of the HER2-enriched subtype and the presence of PIK3CA mutations cannot be well identified). Moreover, the presence of PIK3CA mutations, TILs, and Ki-67 did not have any significantly distinct AUC profiles compared with the presence of HRs (all *p* > 0.05).

### PPVs, NPVs, and LRs

The PPVs, NPVs, and LRs are presented in **Table 1**. Most biomarkers had relatively high NPVs, except HRs. The HER2-enriched subtype consistently had relatively high PPVs, the presence of TILs and HRs had moderate PPVs, and the presence of PIK3CA mutations and Ki-67 had relatively low PPVs. The pooled LRs for each biomarker revealed a significantly higher LR+ value of the HER2-enriched subtype and the presence of TILs than those of the presence of PIK3CA mutations, HRs, and Ki-67 (1.77 and 1.72 *vs*. 1.16, 1.60, and 1.37, respectively). In addition, the LR− value of the HER2-enriched subtype was significantly lower than those for the presence of PIK3CA mutations, TILs, HRs, and Ki-67 (0.54 *vs*. 0.57, 0.79, 0.69, and 0.71, respectively).

**Table 1:** Summary of the pooled sensitivities, specificities, positive and negative predictive values, and likelihood ratios of various biomarkers for predicting pathologically complete response.

Biomarker	Pooled Sensitivity	Pooled Specificity	Pooled PPVs	Pooled NPVs	Pooled Positive LRs	Pooled Negative LRs
HER2-enriched subtype	0.66 (0.63–0.69)	0.62 (0.59–0.64)	0.58 (0.51–0.66)	0.73 (0.67–0.79)	1.77 (1.58–1.98)	0.54 (0.47–0.61)
PIK3CA mutation	0.85 (0.83–0.87)	0.27 (0.25–0.29)	0.37 (0.31–0.43)	0.78 (0.76–0.79)	1.16 (1.12–1.21)	0.57 (0.48–0.67)
TIL	0.49 (0.44–0.54)	0.61 (0.57–0.65)	0.50 (0.36–0.64)	0.71 (0.65–0.78)	1.72 (1.18–2.50)	0.79 (0.69–0.89)
HR	0.54 (0.52–0.57)	0.64 (0.62–0.67)	0.53 (0.46–0.60)	0.64 (0.58–0.71)	1.60 (1.46–1.75)	0.69 (0.63–0.76)
Ki-67	0.68 (0.63–0.73)	0.51 (0.47–0.55)	0.42 (0.29–0.54)	0.76 (0.68–0.84)	1.37 (1.24–1.52)	0.71 (0.55–0.91)

PPV, positive predictive value; NPV, negative predictive value; LR, likelihood ratio; HR, hormone receptor; PIK3CA, phosphatase phosphoinositol-3 (PI3) kinase; TIL, tumor-infiltrating lymphocytes.

All data are reported as a proportion [95% confidence intervals (CI)]. Nonoverlapping 95% CIs suggest statistical significance.

### Pooled Sensitivity and Specificity

**Table 1**, **Figure 4**, and **Supplementary Figure S2** present the pooled sensitivity and specificity values for predicting pCR. The pooled estimates of sensitivity and specificity for the HER2-enriched subtype and the presence of PIK3CA mutations, TILs, HRs, and Ki-67 were 0.66 and 0.62, 0.85 and 0.27, 0.49 and 0.61, 0.54 and 0.64, and 0.68 and 0.51, respectively. The sensitivity of the HER2-enriched subtype was also higher than those of the presence of PIK3CA mutations (*p* < 0.001), TILs (*p* < 0.001), and HRs (*p* < 0.001); the HER2-enriched subtype showed an improved specificity compared with the presence of PIK3CA mutations (*p* < 0.001) and Ki-67 (*p* < 0.001).

**Figure 4:**
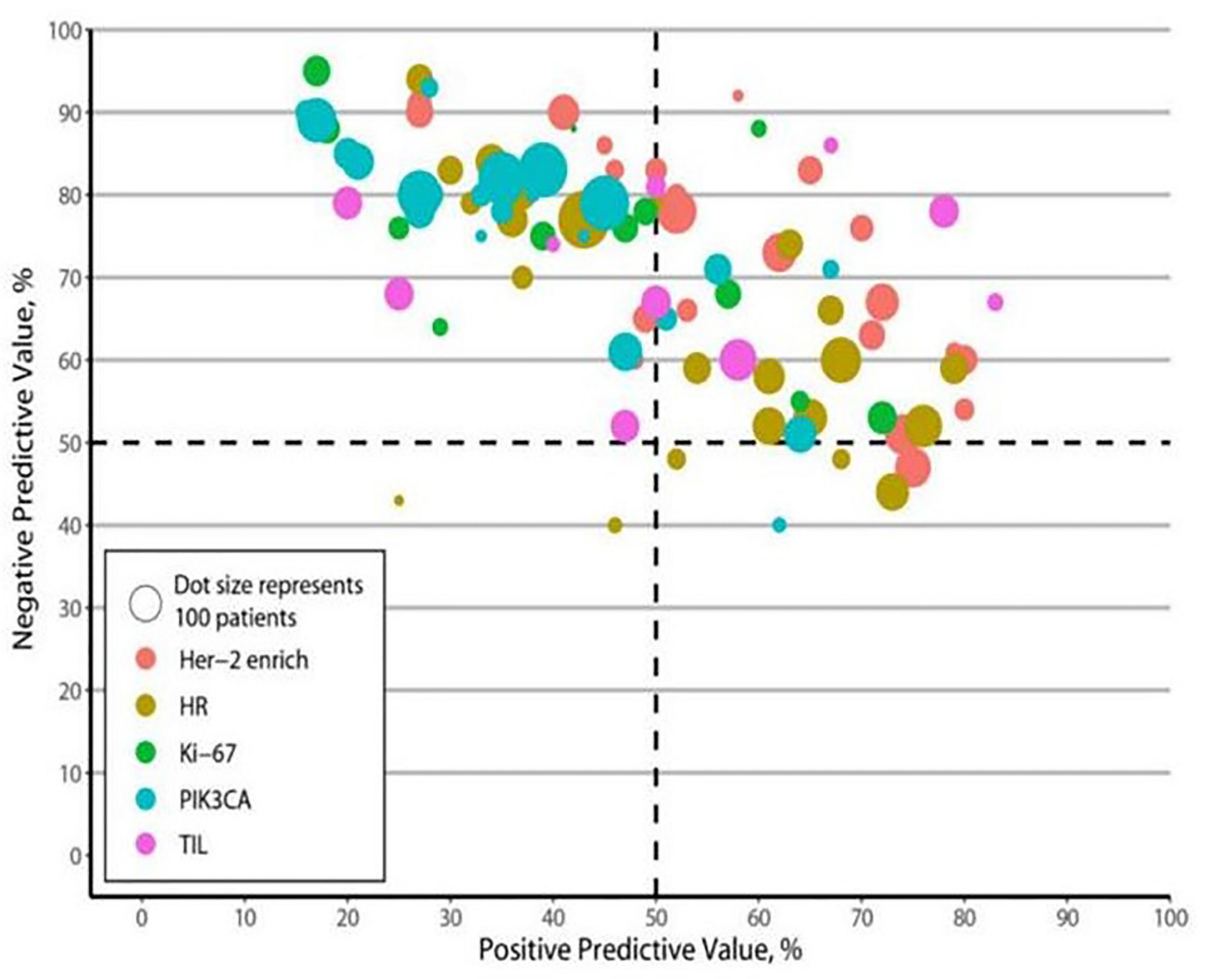
Positive and negative predictive values. HR, hormone receptors; PIK3CA, phosphoinositide 3-kinases; TIL, tumor-infiltrating lymphocytes.

### Publication Bias

**Supplementary Figure S3** shows the results of a Deek’s funnel plot asymmetry test and demonstrates the lack of notable publication biases in the analysis of the HER2-enriched subtype and the presence of PIK3CA mutations, TILs, HRs, and Ki-67 (*p* = 0.48, *p* = 0.25, *p* = 0.42, *p* = 0.72, and *p* = 0.48, respectively).

## Discussion

Suitable biomarkers for predicting the pCR to neoadjuvant therapies with HER2-targeted drugs for the treatment of HER2-positive breast cancer can be used to screen patients who are most likely to benefit from such treatment regimens. An increasing number of studies have identified such biomarkers in recent years. The most reported biomarkers for predicting the pCR rates of HER2-targeted drugs include a HER2-enriched subtype and the presence of PIK3CA mutations, TILs, HRs, and Ki-67. To the best of our knowledge, this meta-analysis is the first to compare the relative diagnostic accuracy of these five biomarkers. Our results revealed that the AUC of the HER2-enriched subtype was significantly higher than those of the presence of PIK3CA mutations, TILs, HRs, and Ki-67. The HER2-enriched subtype also exhibited moderate sensitivity and specificity for predicting pCR and improving LR+ and LR− compared with the other four biomarkers. This meta-analysis documents that the HER2-enriched subtype tends to have moderate diagnostic accuracy for determining pCR to neoadjuvant therapy for HER2-positive breast cancer.

HER2-targeting agents have significantly improved the survival of patients with HER2-positive breast cancer. However, many patients do not respond to these agents; thus, there is an urgent need to explore biomarkers that can screen patients who can benefit from HER2-targeted therapy. Although several studies have been conducted to identify such biomarkers, the validation of these biomarkers has generally failed during randomized clinical trials (91–94). To date, only HER2 has been validated in a clinical setting, although its PPV is low (95). In a neoadjuvant setting, the most reported potential biomarkers include the HER2-enriched subtype and the presence of PIK3CA mutations, TILs, HRs, and Ki-67 (6, 8–10, 24).

Although some previous meta-analyses have shown that the presence of a HER2-enriched subtype, high TIL, and high Ki-67 index predict increased pCR, whereas a PIK3CA mutation and positive HR predict decreased pCR (27–35), the discriminatory diagnostic abilities of these biomarkers remain unclear. In this study, we first comprehensively compared the sensitivity and specificity of the abovementioned five biomarkers. According to the pooled results of the 51 studies we assessed, the sensitivity and specificity of the HER2-enriched subtype were relatively higher than those of other biomarkers. In addition, our AUC analysis estimated the overall diagnostic performance of biomarkers compared with their pooled sensitivity and specificity. We generated an SROC plot by weighting each study based on samples that can further enhance SROC to facilitate reporting (96, 97). The AUC of weighted SROC curves for the HER2-enriched subtype was higher than those of the other biomarkers assessed. Although the difference in AUC values between the HER2-enriched subtype and the presence of PIK3CA mutations was not statistically significant, it was likely due to the wide confidence interval and small samples of PIK3CA mutations. To our knowledge, no consensus on an acceptable AUC value for diagnostic applications has been achieved to date. An AUC value of 0.7–0.8 is considered to represent “satisfactory” diagnostic accuracy (98–100). Therefore, an AUC of 0.71 indicates that a HER2-enriched subtype has moderate diagnostic accuracy.

Considering that AUC values may not be frequently applied in clinical settings and that LRs may be more clinically significant, this meta-analysis also calculated LR+ and LR− values as the measures of diagnostic accuracy (101, 102). A higher LR+ value and a lower LR− value mean that a given parameter has better discriminatory power in contributing to a diagnosis. An LR+ value of >10.0 and LR− value of <0.1 indicate a satisfactory diagnostic test (98). Although the HER2-enriched subtype did not meet these criteria, it had the highest pooled LR+ and lowest pooled LR− values among the five biomarkers assessed. An LR+ value of 1.77 (1.58–1.98) indicates that patients who achieve pCR have 1.77 times greater chances of having a HER2-enriched cancer subtype than those who do not achieve pCR, whereas an LR− value of 0.54 (0.47–0.61) indicates that patients who achieve pCR have a 1.85 times greater chance of having a non-HER2-enriched cancer subtype than those who do not achieve pCR.

Although the HER2-enriched subtype has the best diagnostic accuracy of all biomarkers assessed, the remaining markers still have different degrees of diagnostic accuracy in predicting the pCR rate in HER2-positive breast cancer. Notably, HER2-positive breast cancer is a heterogeneous disease and involves heavy crosstalk among various signaling pathways. On the basis of the distribution of intrinsic breast cancer subtypes, the HER2-enriched subtype comprises approximately 75% of HER2-positive/ER-negative and 30% of HER2-positive/ER-positive tumors and exhibits the characteristic HER2/EGFR pathway activation, high proliferation rate, and immune-activated stroma with elevated TIL levels. In addition, approximately 70% of HER2-positive/ER-positive tumors are luminal subtypes that show low HER2/EGFR pathway activation and a high frequency of PIK3CA mutations (24, 82, 103–105). Phosphorylation of the HER2 kinase domain activates the PI3K/AKT signaling pathway, which is central to a growth-regulating pathway in breast cancer (92, 95). Sustained HR signaling is involved in the escape from HER2 blockade (106). Different biomarkers may be clustered together or are inversely correlated with one another (95). Therefore, the exploration of a combination of HER2-enriched subtypes with multiple biomarkers will provide a direction for future trials focusing on predicting patient responses to therapy.

Our meta-analysis has some limitations. First, all assessed data on the HER2-enriched subtype and the presence of PIK3CA mutations, TILs, and HRs were obtained from prospective trials, whereas most data for Ki-67 were obtained from retrospective studies, as few prospective studies reported these. This might have led to potential bias, although no significant publication bias was found. Second, this meta-analysis was based on study-level but not patient-level data, which might have influenced its precision. Third, clinical and methodological heterogeneity might exist among the studies included, such as variations in the baseline characteristics of patients, treatment regimens, detection methods, and cutoff points for biomarker identification. Finally, the definition of pCR varied across studies. Although most studies defined “ypT0/is ypN0” as pCR, some defined pCR as “ypT0/is”, “ypT0”, and “ypT0 ypN0”. Analyses of subgroups distinguished by varying definitions of pCR were not performed, as most included studies defined “ypT0/is ypN0” as pCR.

## Conclusions

With a broad search strategy and large sample size, this meta-analysis comprehensively analyzed the discriminatory diagnostic ability of a HER2-enriched subtype and the presence of PIK3CA mutations and TILs, HRs, and Ki-67 in predicting pCR to neoadjuvant therapy in patients with HER2-positive breast cancer. The results reveal that the presence of a HER2-enriched subtype has moderate diagnostic accuracy, which is higher than those of the other four biomarkers assessed, although all biomarkers have some degree of diagnostic accuracy. Considering the heterogeneity and the heavy crosstalk among various signaling pathways in HER2-positive breast cancer, combining information about the presence or absence of a HER2-enriched subtype with other biomarkers may help predict patient responses.

## Data Availability Statement

The original contributions presented in the study are included in the article/**Supplementary Material**. Further inquiries can be directed to the corresponding author.

## Author Contributions

FZ and XH contributed equally to this work. FZ, XH, and JZ had full access to all data used/analyzed in the study and take responsibility for the integrity of the data and the accuracy of data analysis. Conceptualization and design of the study: FZ, XH, MW, GS, and JZ. Acquisition, analysis, or interpretation of data: all authors. Drafting of the manuscript: all authors. Critical revision of the manuscript for important intellectual content: GS and JZ. Statistical analysis: FZ, XH, and JZ. Administrative, technical, and material support: FZ, XH, and JZ. Supervision: JZ. All authors contributed to the article and approved the submitted version.

## Funding

This work was supported by grants from the Thousand Talents Program of High-End Innovation of Qinghai Province in China (JZ). The sponsors played no role in the study design, data collection, data analysis, or the decision to submit the article for publication.

## Conflict of Interest

The authors declare that the research was conducted in the absence of any commercial or financial relationships that could be construed as a potential conflict of interest.

## Publisher’s Note

All claims expressed in this article are solely those of the authors and do not necessarily represent those of their affiliated organizations, or those of the publisher, the editors and the reviewers. Any product that may be evaluated in this article, or claim that may be made by its manufacturer, is not guaranteed or endorsed by the publisher.

## Supplementary Material

Supplementary Table 1 Systematic review search strategy.

Supplementary Table 2 Characteristics of the studies included.

Supplementary Figure 1 Risk-of-bias review of each included study.

Supplementary Figure 2 Forest plots of sensitivity and specificity. 2A. Sensitivity. 2B. Specificity.

Supplementary Figure 3 Funnel plot of publication bias.

## References

[1] Slamon DJ Leyland-Jones B Shak S Fuchs H Paton V Bajamonde A . Use of Chemotherapy Plus a Monoclonal Antibody Against HER2 for Metastatic Breast Cancer That Overexpresses HER2. N Engl J Med (2001) 344(11):783–92. doi: 10.1056/NEJM200103153441101 11248153

[2] Slamon DJ Clark GM Wong SG Levin WJ Ullrich A . McGuire WL Human Breast Cancer: Correlation of Relapse and Survival With Amplification of the HER-2/Neu Oncogene. Science (1987) 235:177–82. doi: 10.1126/science.3798106 3798106

[3] Slamon DJ Godolphin W Jones LA Holt JA Wong SG Keith DE . Studies of the HER-2/Neu Proto-Oncogene in Human Breast and Ovarian Cancer. Science (1989) 244:707–12. doi: 10.1126/science.2470152 2470152

[4] Mauri D Pavlidis N Ioannidis JP . Neoadjuvant *Versus* Adjuvant Systemic Treatment in Breast Cancer: A Meta-Analysis. J Natl Cancer Inst (2005) 97:188–94. doi: 10.1093/jnci/dji021 15687361

[5] van der Hage JH van de Velde CJ Mieog JS . Preoperative Chemotherapy for Women With Operable Breast Cancer. Cochrane Database Syst Rev (2007) 2:CD005002. doi: 10.1002/1465185.CD005002 PMC7388837 17443564

[6] Choong GM Cullen GD O’Sullivan C . Evolving Standards of Care and New Challenges in the Management of HER2-Positive Breast Cancer. CA. A Cancer J Clin (2020) 70(5):355–74. doi: 10.3322/caac.21634 32813307

[7] Network NCC . NCCN Clinical Practice Guidelines in Oncolgy (NCCN Guidelines) Breast Cancer NCCN Evidence Blocks (Version 7. 2021).

[8] Wuerstlein R Harbeck N . Neoadjuvant Therapy for HER2-Positive Breast Cancer. Rev Recent Clin Trials (2017) 12:81–92. doi: 10.2174/1574887112666170202165049 28164759

[9] Dent S Oyan B Honig A Mano M Howell S . HER2-Targeted Therapy in Breast Cancer: A Systematic Review of Neoadjuvant Trials. Cancer Treat Rev (2013) 39:622–31. doi: 10.1016/j.ctrv.2013.01.002 23434074

[10] Pernas S Tolaney SM . HER2-Positive Breast Cancer: New Therapeutic Q22 Frontiers and Overcoming Resistance. Ther Adv Med Oncol (2019) 443-63. 11:1758835919833519. doi: 10.1177/1758835919833519 PMC6425535 30911337

[11] von Minckwitz G Untch M Blohmer JU Costa SD Eidtmann H Fasching PA . Definition and Impact of Pathologic Complete Response on Prognosis After Neoadjuvant Chemotherapy in Various Intrinsic Breast Cancer Subtypes. J Clin Oncol (2012) 30:1796–804. doi: 10.1200/JCO.2011.38.8595 22508812

[12] Broglio KR Quintana M Foster M Olinger M McGlothlin A Berry SM . Association of Pathologic Complete Response to Neoadjuvant Therapy in HER2-Positive Breast Cancer With Long-Term Outcomes: A Meta-Analysis. JAMA Oncol (2016) 2:751–60. doi: 10.1001/jamaoncol.2015.6113 26914222

[13] Spring LM Fell G Arfe A Sharma C Greenup R Reynolds KL . Pathologic Complete Response After Neoadjuvant Chemotherapy and Impact on Breast Cancer Recurrence and Survival: A Comprehensive Meta-Analysis. Clin Cancer Res (2020) 26:2838–48. doi: 10.1158/1078-0432.CCR-19-3492 PMC7299787 32046998

[14] Perou CM Sørlie T Eisen MB van de Rijn M Jeffrey SS Rees CA . Molecular Portraits of Human Breast Tumours. Nature (2000) 406(6797):747–52. doi: 10.1038/35021093 10963602

[15] Parker JS Mullins M Cheang MC Leung S Voduc D Vickery T . Supervised Risk Predictor of Breast Cancer Based on Intrinsic Subtypes. J Clin Oncol (2009) 27(8):1160–7. doi: 10.1200/JCO.2008.18.1370 PMC2667820 19204204

[16] Prat A Perou CM . Deconstructing the Molecular Portraits of Breast Cancer. Mol Oncol (2011) 5:5–23. doi: 10.1016/j.molonc.2010.11.003 21147047 PMC5528267

[17] Prat A Pascual T De Angelis C Gutierrez C Llombart-Cussac A Wang T . HER2-Enriched Subtype and ERBB2 Expression in HER2-Positive Breast Cancer Treated With Dual HER2Blockade. J Natl Cancer Inst (2020) 112:46–54. doi: 10.1093/jnci/djz042 31037288 PMC7850037

[18] Cancer Genome Atlas Network . Comprehensive Molecular Portraits of Human Breast Tumours. Nature (2012) 490:61–70. doi: 10.1038/nature11412 23000897 PMC3465532

[19] Franke TF Kaplan DR Cantley LC Toker A . Direct Regulation of the Akt Proto-Oncogene Product by Phosphatidylinositol-3,4-Bisphosphate. Science (1997) 275:665–8. doi: 10.1126/science.275.5300.665 9005852

[20] Berns K Horlings HM Hennessy BT Madiredjo M Hijmans EM Beelen K . A Functional Genetic Approach Identifies the PI3K Pathway as a Major Determinant of Trastuzumab Resistance in Breast Cancer. Cancer Cell (2007) 12(4):395–402. doi: 10.1016/j.ccr.2007.08.030 17936563

[21] Hortobagyi GN Chen D Piccart M Rugo HS Burris HA 3rd Pritchard KI . Correlative Analysis of Genetic Alterations and Everolimus Benefit in Hormone Receptor-Positive, Human Epidermal Growth Factor Receptor 2-Negative Advanced Breast Cancer: Results From BOLERO-2. J Clin Oncol (2016) 34(5):419–26. doi: 10.1200/JCO.2014.60.1971. Published correction appears in J Clin Oncol. 2019 Feb 1;37(4):354] [published correction appears in J Clin Oncol. 2019 Feb 1;37(4):355. PMC5070556 26503204

[22] Bianchini G Gianni L . The Immune System and Response to HER2-Targeted Treatment in Breast Cancer. Lancet Oncol (2014) 15:e58–68. doi: 10.1016/S1470-2045(13)70477-7 24480556

[23] André F O'Regan R Ozguroglu M Toi M Xu B Jerusalem G . Everolimus for Women With Trastuzumab-Resistant, HER2-Positive, Advanced Breast Cancer (BOLERO-3): A Randomised, Double-Blind, Placebo-Controlled Phase 3 Trial. Lancet Oncol (2014) 15(6):580–91. doi: 10.1016/S1470-2045(14)70138-X 24742739

[24] Brandão M Caparica R Malorni L Prat A Carey LA Piccart M . What Is the Real Impact of Estrogen Receptor Status on the Prognosis and Treatment of HER2-Positive Early Breast Cancer? Clin Cancer Res (2020) 26:2783–8. doi: 10.1158/1078-0432.CCR-19-2612 PMC8324078 32046997

[25] Schettini F Buono G Cardalesi C Desideri I De Placido S Del Mastro L . Hormone Receptor/Human Epidermal Growth Factor Receptor 2-Positive Breast Cancer: Where We Are Now and Where We Are Going. Cancer Treat Rev (2016) 46:20–6. doi: 10.1016/j.ctrv.2016.03.012 27057657

[26] Brown JR DiGiovanna MP Killelea B Lannin DR Rimm DL . Quantitative Assessment Ki-67Score for Prediction of Response to Neoadjuvant Chemotherapy in Breast Cancer. Lab Invest (2014) 94:98–106. doi: 10.1038/labinvest.2013.128 24189270

[27] Schettini F Pascual T Conte B Chic N Brasó-Maristany F Galván P . HER2-Enriched Subtype and Pathological Complete Response in HER2-Positive Breast Cancer: A Systematic Review and Meta-Analysis. Cancer Treat Rev (2020) 84:101965. doi: 10.1016/j.ctrv.2020.101965 32000054 PMC7230134

[28] Houssami N Macaskill P von Minckwitz G Marinovich ML Mamounas E . Meta-Analysis of the Association of Breast Cancer Subtype and Pathologic Complete Response to Neoadjuvant Chemotherapy. Eur J Cancer (2012) 48:3342–54. doi: 10.1016/j.ejca.2012.05.023 22766518

[29] Zhao B Zhao H . Impact of Clinicopathological Characteristics on the Efficacy of Neoadjuvant Therapy in Patients With Human Epidermal Growth Factor Receptor-2-Positive Breast Cancer. Int J Cancer (2018) 142:844–53. doi: 10.1002/ijc.31097 29023765

[30] Fan H Li C Xiang Q Xu L Zhang Z Liu Q . Mutations and Their Response to Neoadjuvant Treatment in Early Breast Cancer: A Systematic Review and Meta-Analysis. Thorac Cancer (2018) 9:571–9. doi: 10.1111/1759-7714.12618 PMC5928352 29575819

[31] Ibrahim EM Kazkaz GA Al-Mansour MM Al-Foheidi ME . The Predictive and Prognostic Role of Phosphatase Phosphoinositol-3 (PI3) Kinase (PIK3CA) Mutation in HER2-Positive Breast Cancer Receiving HER2-Targeted Therapy: A Meta-Analysis. Breast Cancer Res Treat (2015) 152:463–76. doi: 10.1007/s10549-015-3480-6 26105797

[32] Xu T He BS Liu XX Hu XX Lin K Pan YQ . The Predictive and Prognostic Role of Stromal Tumor-Infiltrating Lymphocytes in HER2-Positive Breast Cancer With Trastuzumab-Based Treatment: A Meta-Analysis and Systematic Review. J Cancer (2017) 8:3838–48. doi: 10.7150/jca.21051 PMC5688937 29151971

[33] Solinas C Ceppi M Lambertini M Scartozzi M Buisseret L Garaud S . Tumor-Infiltrating Lymphocytes in Patients With HER2-Positive Breast Cancer Treated With Neoadjuvant Chemotherapy Plus Trastuzumab, Lapatinib or Their Combination: A Meta-Analysis of Randomized Controlled Trials. Cancer Treat Rev (2017) 57:8–15. doi: 10.1016/j.ctrv.2017.04.005 28525810

[34] Tao M Chen S Zhang X Zhou Q . Ki-67 Labeling Index Is a Predictive Markerfor a Pathological Complete Response to Neoadjuvant Chemotherapy in Breast Cancer: A Meta-Analysis. Med (Baltimore) (2017) 96:e9384. doi: 10.1097/MD.0000000000009384 PMC5758242 29390540

[35] Chen X He C Han D Zhou M Wang Q Tian J . The Predictive Value of Ki-67 Before Neoadjuvant Chemotherapy for Breast Cancer: A Systematic Review and Meta-Analysis. Future Oncol (2017) 13:843–57. doi: 10.2217/fon-2016-0420 28075166

[36] Moher D Liberati A Tetzlaff J . Altman DGPRISMA Group. Preferred Reporting Items for Systematic Reviews and Meta-Analyses: The PRISMA Statement. PloS Med (2009) 6(7):e1000097. doi: 10.1371/journal.pmed.1000097 19621072 PMC2707599

[37] Whiting P Rutjes AW Reitsma JB Bossuyt PM Kleijnen J . The Development of QUADAS: A Tool for the Quality Assessment of Studies of Diagnostic Accuracy Included in Systematic Reviews. BMC Med Res Methodol (2003) 3:25. doi: 10.1186/1471-2288-3-25 14606960 PMC305345

[38] Whiting PF Weswood ME Rutjes AW Reitsma JB Bossuyt PN Kleijnen J . Evaluation of QUADAS, a Tool for the Quality Assessment of Diagnostic Accuracy Studies. BMC Med Res Methodol (2006) 6:9. doi: 10.1186/1471-2288-6-9 16519814 PMC1421422

[39] Whiting PF Rutjes AW Westwood ME Mallett S Deeks JJ Reitsma JB . QUADAS-2: A Revised Tool for the Quality Assessment of Diagnostic Accuracy Studies. Ann Intern Med (2011) 155:529–36. doi: 10.7326/0003-4819-155-8-201110180-00009 22007046

[40] Rimawi MF Mayer IA Forero A Nanda R Goetz MP Rodriguez AA . Multicenter Phase II Study of Neoadjuvant Lapatinib and Trastuzumab With Hormonal Therapy and Without Chemotherapy in Patients With Human Epidermal Growth Factor Receptor 2-Overexpressing Breast Cancer: TBCRC 006. J Clin Oncol (2013) 31:1726–3. doi: 10.1200/JCO.2012.44.8027 PMC3641695 23569315

[41] Rimawi MF Niravath P Wang T Rexer BN Forero A Wolff AC . TBCRC023: A Randomized Phase II Neoadjuvant Trial of Lapatinib Plus Trastuzumab Without Chemotherapy for 12 *Versus* 24 Weeks in Patients With HER2-Positive Breast Cancer. Clin. Cancer Res (2020) 26:821–7. doi: 10.1158/1078-0432.CCR-19-0851 31662331

[42] Llombart-Cussac A Cortés J Paré L Galván P Bermejo B Martínez N . HER2-Enriched Subtype as a Predictor of Pathological Complete Response Following Trastuzumab and Lapatinib Without Chemotherapy in Early-Stage HER2-Positive Breast Cancer (PAMELA): An Open-Label, Single-Group, Multicentre, Phase 2 Trial. Lancet Oncol (2017) 18:545–54. doi: 10.1016/S1470-2045(17)30021-9 28238593

[43] Guarneri V Dieci MV Bisagni G Frassoldati A Bianchi GV De Salvo GL . De-Escalated Therapy for HR+/HER2+ Breast Cancer Patients With Ki67 Response After 2-Week Letrozole: Results of the PerELISA Neoadjuvant Study. Ann Oncol (2019) 30:921–6. doi: 10.1093/annonc/mdz055 PMC6594455 30778520

[44] Gavilá J Oliveira M Pascual T Perez-Garcia J Gonzàlez X Canes J . Safety, Activity, and Molecular Heterogeneity Following Neoadjuvant Non-Pegylated Liposomal Doxorubicin, Paclitaxel, Trastuzumab, and Pertuzumab in HER2-Positive Breast Cancer (Opti-HER HEART): An Open-Label, Single-Group, Multicenter, Phase 2 Trial. BMC Med (2019) 17:8. doi: 10.1186/s12916-018-1233-1 30621698 PMC6325829

[45] de Azambuja E Holmes AP Piccart-Gebhart M Holmes E Di Cosimo S Swaby RF . Lapatinib With Trastuzumab for HER2-Positive Early Breast Cancer (NeoALTTO): Survival Outcomes of a Randomised, Open-Label, Multicentre, Phase 3 Trial and Their Association With Pathological Complete Response. Lancet Oncol (2014) 15:1137–46. doi: 10.1016/S1470-2045(14)70320-1 25130998

[46] Dieci MV Prat A Tagliafico E Paré L Ficarra G Bisagni G . Integrated Evaluation of PAM50 Subtypes and Immune Modulation of pCR in HER2-Positive Breast Cancer Patients Treated With Chemotherapy and HER2-Targeted Agents in the CherLOB Trial. Ann Oncol (2016) 27:1867–73. doi: 10.1093/annonc/mdw262 27484801

[47] Swain SM Ewer MS Viale G Delaloge S Ferrero JM Verrill M . Pertuzumab, Trastuzumab, and Standard Anthracycline- and Taxane-Based Chemotherapy for the Neoadjuvant Treatment of Patients With HER2-Positive Localized Breast Cancer (BERENICE): A Phase II, Open-Label, Multicenter, Multinational Cardiac Safety Study. Ann Oncol (2018) 29:646–53. doi: 10.1093/annonc/mdx773 PMC5888999 29253081

[48] Bayraktar S Royce M Stork-Sloots L de Snoo F Glück S . Molecular Subtyping Predicts Pathologic Tumor Response in Early-Stage Breast Cancer Treated With Neoadjuvant Docetaxel Plus Capecitabine With or Without Trastuzumab Chemotherapy. Med Oncol (2014) 31:163. doi: 10.1007/s12032-014-0163-9 25186065

[49] Swain SM Tang G Lucas PC Robidoux A Goerlitz D Harris BT . Pathologic Complete Response and Outcomes by Intrinsic Subtypes in NSABP B-41, a Randomized Neoadjuvant Trial of Chemotherapy With Trastuzumab, Lapatinib, or the Combination. Breast Cancer Res Treat (2019) 178:389–99. doi: 10.1007/s10549-019-05398-3 PMC6797698 31428908

[50] Prat A Bianchini G Thomas M Belousov A Cheang MC Koehler A . Research-Based PAM50 Subtype Predictor Identifies Higher Responses and Improved Survival Outcomes in HER2-Positive Breast Cancer in the NOAH Study. Clin Cancer Res (2014) 20:511–21. doi: 10.1158/1078-0432.CCR-13-0239 24443618

[51] Prat A Fan C Fernández A Hoadley KA Martinello R Vidal M . Response and Survival of Breast Cancer Intrinsic Subtypes Following Multi-Agent Neoadjuvant Chemotherapy. BMC Med (2015) 13:303. doi: 10.1186/s12916-015-0540-z 26684470 PMC4683815

[52] Prat A Slamon D Hurvitz SA Press MF Phillips GL Valverde VL . Abstract PD3-06: Association of Intrinsic Subtypes With Pathological Complete Response (pCR) in the KRISTINE Neoadjuvant Phase 3 Clinical Trial in HER2-Positive Early Breast Cancer (EBC). Cancer Res (2018) 78(4):PD3–06. doi: 10.1158/1538-7445.SABCS17-PD3-06

[53] Nakatsukasa K Koyama H Oouchi Y Imanishi S Mizuta N Sakaguchi K . Docetaxel, Cyclophosphamide, and Trastuzumab as Neoadjuvant Chemotherapy for HER2-Positive Primary Breast Cancer. Breast Cancer (2017) 24:92–7. doi: 10.1007/s12282-016-0677-4 26874836

[54] Carey LA Berry DA Cirrincione CT Barry WT Pitcher BN Harris LN . Molecular Heterogeneity and Response to Neoadjuvant Human Epidermal Growth Factor Receptor 2 Targeting in CALGB 40601, a Randomized Phase III Trial of Paclitaxel Plus Trastuzumab With or Without Lapatinib. J Clin Oncol (2016) 34:542–9. doi: 10.1200/JCO.2015.62.1268 PMC4980567 26527775

[55] Lesurf R Griffith OL Griffith M Hundal J Trani L Watson MA . Genomic Characterization of HER2-Positive Breast Cancer and Response to Neoadjuvant Trastuzumab and Chemotherapy-Results From the ACOSOG Z1041 (Alliance) Trial. Ann Oncol (2017) 28:1070–7. doi: 10.1093/annonc/mdx048 PMC5790063 28453704

[56] Bonnefoi H Jacot W Saghatchian M Moldovan C Venat-Bouvet L Zaman K . Neoadjuvant Treatment With Docetaxel Plus Lapatinib, Trastuzumab, or Both Followed by an Anthracycline-Based Chemotherapy in HER2-Positive Breast Cancer: Results of the Randomised Phase II EORTC 10054 Study. Ann Oncol (2015) 26(2):325–32. doi: 10.1093/annonc/mdu551 PMC4304382 25467016

[57] Untch M Rezai M Loibl S Fasching PA Huober J Tesch H . Neoadjuvant Treatment With Trastuzumab in HER2-Positive Breast Cancer: Results From the GeparQuattro Study. J Clin Oncol y (2010) 28(12):2024–31. doi: 10.1200/JCO.2009.23.8451 20308670

[58] Alba E Albanell J de la Haba J Barnadas A Calvo L Sánchez-Rovira P . Trastuzumab or Lapatinib With Standard Chemotherapy for HER2-Positive Breast Cancer: Results From the GEICAM/2006-14 Trial. Br J Cancer (2014) 110(5):1139–47. doi: 10.1038/bjc.2013.831 PMC3950860 24457911

[59] Holmes FA Espina V Liotta LA Nagarwala YM Danso M McIntyre KJ . Pathologic Complete Response After Preoperative Anti-HER2 Therapy Correlates With Alterations in PTEN, FOXO, Phosphorylated Stat5, and Autophagy Protein Signaling. BMC Res Notes (2013) 6(1):507. doi: 10.1186/1756-0500-6-507 24304724 PMC3915616

[60] Baselga J Bradbury I Eidtmann H Di Cosimo S de Azambuja E Aura C . Lapatinib With Trastuzumab for HER2-Positive Early Breast Cancer (NeoALTTO): A Randomised, OpenLabel, Multicentre, Phase 3 Trial. Lancet (9816) 2012:633–40. doi: 10.1016/S0140-6736(11)61847-3 PMC5705192 22257673

[61] Gianni L Pienkowski T Im YH Roman L Tseng LM Liu MC . Efficacy and Safety of Neoadjuvant Pertuzumab and Trastuzumab in Women With Locally Advanced, Inflammatory, or Early HER2-Positive Breast Cancer (NeoSphere): A Randomised Multicentre, Open-Label, Phase 2 Trial. Lancet Onco (2012) 13(1):25–32. doi: 10.1016/S1470-2045(11)70336-9 22153890

[62] Robidoux A Tang G Rastogi P Geyer CE Jr Azar CA Atkins JN . Lapatinib as a Component of Neoadjuvant Therapy for HER2-Positive Operable Breast Cancer (NSABP Protocol B-41): An Open-Label, Randomised Phase 3 Trial. Lancet Onco (2013) 14(12):1183–92. doi: 10.1016/S1470-2045(13)70411-X 24095300

[63] Schneeweiss A Chia S Hickish T Harvey V Eniu A Hegg R . Pertuzumab Plus Trastuzumab in Combination With Standard Neoadjuvant Anthracycline-Containing and Anthracycline-Free Chemotherapy Regimens in Patients With HER2-Positive Early Breast Cancer: A Randomized Phase II Cardiac Safety Study (TRYPHAENA). Ann Oncol (2013) 24(9):2278–84. doi: 10.1093/annonc/mdt182 23704196

[64] Pierga JY P Delaloge S Espié M Brain E Sigal-Zafrani B Mathieu MC . A Multicenter Randomized Phase II Study of Sequential Epirubicin/Cyclophosphamide Followed by Docetaxel With or Without Celecoxib or Trastuzumab According to HER2 Status, as Primary Chemotherapy for Localized Invasive Breast Cancer Patients. Breast Cancer Res Trea (2010) 122(2):429–37. doi: 10.1007/s10549-010-0939-3 20480225

[65] Patel TA Ensor JE Creamer SL Boone T Rodriguez AA Niravath PA . A Randomized, Controlled Phase II Trial of Neoadjuvant Ado-Trastuzumab Emtansine, Lapatinib, and NabPaclitaxel *Versus* Trastuzumab, Pertuzumab, and Paclitaxel in HER2-Positive Breast Cancer (TEAL Study). Breast Cancer Res Trea (2019) 21(1):1–9. doi: 10.1186/s13058-019-1186-0 PMC6720931 31477168

[66] Yee D DeMichele AM Yau C Isaacs C Symmans WF Albain KS . Association of Event-Free and Distant Recurrence–Free Survival With Individual-Level Pathologic Complete Response in Neoadjuvant Treatment of Stages 2 and 3 Breast Cancer: Three-Year Follow-Up Analysis for the I-SPY2 Adaptively Randomized Clinical Trial. JAMA Oncol (2020) 6(9):1355–62. doi: 10.1001/jamaoncol.2020.2535 PMC7378873 32701140

[67] Esserman LJ Berry DA DeMichele A Carey L Davis SE Buxton M . Pathologic Complete Response Predicts Recurrence-Free Survival More Effectively by Cancer Subset: Results From the I-SPY 1 TRIAL—CALGB 150007/150012, ACRIN 6657. J Clin Oncol (2012) 30(26):3242. doi: 10.1200/JCO.2011.39.2779 22649152 PMC3434983

[68] Saracchini S Foltran L Tuccia F Bassini A Sulfaro S Micheli E . Phase II Study of LiposomeEncapsulated Doxorubicin Plus Cyclophosphamide, Followed by Sequential Trastuzumab Plus Docetaxel as Primary Systemic Therapy for Breast Cancer Patients With HER2 Overexpression or Amplification. Breast (2013) 22(6):1101–7. doi: 10.1016/j.breast.2013.09.001 24074879

[69] Kurozumi S Inoue K Takei H Matsumoto H Kurosumi M Horiguchi J . ER, PgR, Ki67, P27 Kip1, and Histological Grade as Predictors of Pathological Complete Response in Patients With HER2-Positive Breast Cancer Receiving Neoadjuvant Chemotherapy Using Taxanes Followed by Fluorouracil, Epirubicin, and Cyclophosphamide Concomitant With Trastuzumab. BMC Cancer (2015) 15(1):1–8. doi: 10.1186/s12885-015-1641-y 26345461 PMC4562359

[70] Huang L Chen T Chen C Chen S Liu Y Wu J . Prognostic and Predictive Value of Phospho-P44/42 and pAKT in HER2-Positive Locally Advanced Breast Cancer Patients Treated With Anthracycline-Based Neoadjuvant Chemotherapy. World J Surg Oncol (2013) 11(1):307. doi: 10.1186/1477-7819-11-307 24289519 PMC4220778

[71] Zhang GC Qian XK Guo ZB Ren CY Yao M Li XR . Pre-Treatment Hormonal Receptor Status and Ki67 Index Predict Pathologic Complete Response to Neoadjuvant Trastuzumab/Taxanes But Not Disease-Free Survival in HER2-Positive Breast Cancer Patients. Med Oncol (2012) 29(5):3222–31. doi: 10.1007/s12032-012-0242-8 22547076

[72] Alba E Lluch A Ribelles N Anton-Torres A Sanchez-Rovira P Albanell J . High Proliferation Predicts Pathological Complete Response to Neoadjuvant Chemotherapy in Early Breast Cancer. Oncologist (2016) 21(2):150. doi: 10.1634/theoncologist.2015-0312 26786263 PMC4746087

[73] Bria E Furlanetto J Carbognin L Brunelli M Caliolo C Nortilli R . Human Epidermal Growth Factor Receptor 2–Positive Breast Cancer: Heat Shock Protein 90 Overexpression, Ki67 Proliferative Index, and Topoisomerase II-a Co-Amplification as Predictors of Pathologic Complete Response to Neoadjuvant Chemotherapy With Trastuzumab and Docetaxel. Clin Breast Cancer (2015) 15(1):16–23. doi: 10.1016/j.clbc.2014.05.004 25034441

[74] Kim KI Lee KH Kim TR Chun YS Lee TH Park HK . Predictor of Response to Neoadjuvant Chemotherapy in Breast Cancer Patients. J Breast Cancer (2014) 17(1):40–6. doi: 10.4048/jbc.2014.17.1.40 PMC3988341 24744796

[75] Ding J Yang Y Jiang L Wu W Shao Z . Predictive Factors of Pathologic Complete Response in HER2-Positive and Axillary Lymph Node Positive Breast Cancer After Neoadjuvant Paclitaxel, Carboplatin Plus With Trastuzumab. Oncotarget (2017) 8:56626–34. doi: 10.18632/oncotarget.17993 PMC5593588 28915617

[76] Harbeck N Gluz O Christgen M Kates RE Braun M Küemmel S . De-Escalation Strategies in Human Epidermal Growth Factor Receptor 2 (HER2)–Positive Early Breast Cancer (BC): Final Analysis of the West German Study Group Adjuvant Dynamic Marker-Adjusted Personalized Therapy Trial Optimizing Risk Assessment and Therapy Response Prediction in Early BC HER2-And Hormone Receptor–Positive Phase II Randomized Trial—Efficacy, Safety, and Predictive Markers for 12 Weeks of Neoadjuvant Trastuzumab Emtansine With or Without Endocrine Therapy (ET) *Versus* Trastuzumab Plus Et. J Clin Oncol (2017) 35(26):3046–54. doi: 10.1200/JCO.2016.71.9815 28682681

[77] Denkert C von Minckwitz G Brase JC Sinn BV Gade S Kronenwett R . Tumor-Infiltrating Lymphocytes and Response to Neoadjuvant Chemotherapy With or Without Carboplatin in Human Epidermal Growth Factor Receptor 2-Positive and Triple-Negative Primary Breast Cancers. J Clin Oncol (2015) 33(9):983–91. doi: 10.1200/JCO.2014.58.1967 25534375

[78] Salgado R Denkert C Campbell C Savas P Nuciforo P Aura C . Tumor-Infiltrating Lymphocytes and Associations With Pathological Complete Response and Event-Free Survival in HER2-Positive Early-Stage Breast Cancer Treated With Lapatinib and Trastuzumab: A Secondary Analysis of the NeoALTTO Trial. JAMA Oncol (2015) 1(4):448–55. doi: 10.1001/jamaoncol.2015.0830 PMC5551492 26181252

[79] Ingold Heppner B Untch M Denkert C Pfitzner BM Lederer B Schmitt W . Tumor-Infiltrating Lymphocytes: A Predictive and Prognostic Biomarker in Neoadjuvant-Treated HER2-Positive Breast Cancer. Clin Cancer Res (2016) 22(23):5747–54. doi: 10.1158/1078-0432.CCR-15-2338 27189162

[80] De Angelis C Nagi C Hoyt CC Liu L Roman K Wang C . Evaluation of the Predictive Role of Tumor Immune Infiltrate in Patients With HER2-Positive Breast Cancer Treated With Neoadjuvant Anti-HER2 Therapy Without Chemotherapy. Clin Cancer Res (2020) 26(3):738–45. doi: 10.1158/1078-0432.CCR-19-1402 PMC7002194 31653641

[81] Loibl S Majewski I Guarneri V Nekljudova V Holmes E Bria E . PIK3CA Mutations Are Associated With Reduced Pathological Complete Response Rates in Primary HER2- Positive Breast Cancer: Pooled Analysis of 967 Patients From Five Prospective Trials Investigating Lapatinib and Trastuzumab. Ann Oncol (2016) 27(8):1519–25. doi: 10.1093/annonc/mdw197 PMC6279074 27177864

[82] Bianchini G Kiermaier A Bianchi GV Im YH Pienkowski T Liu MC . Biomarker Analysis of the NeoSphere Study: Pertuzumab, Trastuzumab, and Docetaxel *Versus* Trastuzumab Plus Docetaxel, Pertuzumab Plus Trastuzumab, or Pertuzumab Plus Docetaxel for the Neoadjuvant Treatment of HER2-Positive Breast Cancer. Breast Cancer Res (2017) 19(1):1–12. doi: 10.1186/s13058-017-0806-9 28183321 PMC5299741

[83] Dave B Migliaccio I Gutierrez MC Wu MF Chamness GC Wong H . Loss of Phosphatase and Tensin Homolog or Phosphoinositol-3 Kinase Activation and Response to Trastuzumab or Lapatinib in Human Epidermal Growth Factor Receptor 2–Overexpressing Locally Advanced Breast Cancers. J Clin Oncol (2011) 29(2):166. doi: 10.1200/JCO.2009.27.7814 21135276 PMC3058274

[84] Harbeck N Gluz O Christgen M Braun M Kuemmel S Schumacher C . Abstract S5-03: Final Analysis of WSG-ADAPT HER2+/HR+ Phase II Trial: Efficacy, Safety, and Predictive Markers for 12-Weeks of Neoadjuvant TDM1 With or Without Endocrine Therapy Versus Trastuzumab+ Endocrine Therapy in HER2-Positive Hormone-Receptor-Positive Early Breast Cancer. San Antonio, TX (2016).

[85] Sueta A Yamamoto Y Yamamoto-Ibusuki M Hayashi M Takeshita T Yamamoto S . An Integrative Analysis of PIK3CA Mutation, PTEN, and INPP4B Expression in Terms of Trastuzumab Efficacy in HER2-Positive Breast Cancer. PloS One (2014) 9(12):e116054. doi: 10.1371/journal.pone.0116054 25542038 PMC4277449

[86] Loibl S de la Pena L Nekljudova V Zardavas D Michiels S Denkert C . Neoadjuvant Buparlisib Plus Trastuzumab and Paclitaxel for Women With HER2+ Primary Breast Cancer: A Randomised, Double-Blind, Placebo-Controlled Phase II Trial (NeoPHOEBE). Eur J Cancer (2017) 85:133–45. doi: 10.1016/j.ejca.2017.08.020 PMC5640494 28923573

[87] Rimawi MF De Angelis C Contreras A Pareja F Geyer FC Burke KA . Low PTEN Levels and PIK3CA Mutations Predict Resistance to Neoadjuvant Lapatinib and Trastuzumab Without Chemotherapy in Patients With HER2 OverExpressing Breast Cancer. Breast Cancer Res Treat (2018) 167(3):731–40. doi: 10.1007/s10549-017-4533-9 PMC5821069 29110152

[88] Guarneri V Dieci MV Carbognin L Maiorana A Bettelli S Tortora G . Activity of Neoadjuvant Lapatinib (L) Plus Trastuzumab (T) for Early Breast Cancer (EBC) According to PIK3CA Mutations: Pathological Complete Response (pCR) Rate in the CherLOB Study and Pooled Analysis of Randomized Trials. Ann Oncol (2014) 25:iv85. doi: 10.1093/annonc/mdu327.2

[89] Schneeweiss A Chia S Hegg R Tausch C Deb R Ratnayake J . Evaluating the Predictive Value of Biomarkers for Efficacy Outcomes in Response to Pertuzumab-and Trastuzumab-Based Therapy: An Exploratory Analysis of the TRYPHAENA Study. Breast Cancer Res (2014) 16(4):1–12. doi: 10.1186/bcr3690 PMC4226982 25005255

[90] Majewski IJ Nuciforo P Mittempergher L Bosma AJ Eidtmann H Holmes E . PIK3CA Mutations Are Associated With Decreased Benefit to Neoadjuvant Human Epidermal Growth Factor Receptor 2–Targeted Therapies in Breast Cancer. J Clin Oncol (2015) 33(12):1334. doi: 10.1200/JCO.2014.55.2158 25559818 PMC5087318

[91] Goutsouliak K Veeraraghavan J Sethunath V De Angelis C Osborne CK Rimawi MF . Towards Personalized Treatment for Early Stage HER2-Positive Breast Cancer. Nat Rev Clin Oncol (2019), 1–18. doi: 10.1038/s41571-019-0299-9 31836877 PMC8023395

[92] Nicolini A Ferrari P Duffy MJ . Prognostic and Predictive Biomarkers in Breast Cancer: Past, Present and Future[C]//Seminars. In: Cancer Biology, vol. Vol. 52. . Italy: Academic Press (2018). p. 56–73. 10.1016/j.semcancer.2017.08.010 28882552

[93] Triulzi T Bianchi GV Tagliabue E . Predictive Biomarkers in the Treatment of HER2-Positive Breast Cancer: An Ongoing Challenge. Future Oncol (2016) 12(11):1413–28. doi: 10.2217/fon-2015-0025 27007660

[94] Di Modica M Tagliabue E Triulzi T . Predicting the Efficacy of HER2-Targeted Therapies: A Look at the Host. Dis Markers (2017) 2017:7849108. doi: 10.1155/2017/7849108 29403144 PMC5748305

[95] Gingras I Gebhart G de Azambuja E Piccart-Gebhart M . HER2-Positive Breast Cancer Is Lost in Translation: Time for Patient-Centered Research. Nat Rev Clin Oncol (2017) 14(11):669. doi: 10.1038/nrclinonc.2017.96 28762384

[96] Patel A Cooper N Freeman S Sutton A . Graphical Enhancements to Summary Receiver Operating Characteristic Plots to Facilitate the Analysis and Reporting of Meta-Analysis of Diagnostic Test Accuracy Data. Res Synth Methods (2020) 12(1):34–44. doi: 10.1002/jrsm.1439 32706182

[97] Lu S Stein JE Rimm DL Wang DW Bell JM Johnson DB . Comparison of Biomarker Modalities for Predicting Response to PD-1/PD-L1 Checkpoint Blockade: A Systematic Review and Meta-Analysis. JAMA Oncol (2019) 5(8):1195–204. doi: 10.1001/jamaoncol.2019.1549 PMC6646995 31318407

[98] Šimundić AM . Measures of diagnostic accuracy: basic definitions. Ejifcc (2009) 19(4):203. 27683318 PMC4975285

[99] Frati A Chereau E Coutant C Bezu C Antoine M Chopier J . Comparison of Two Nomograms to Predict Pathologic Complete Responses to Neoadjuvant Chemotherapy for Breast Cancer: Evidence That HER2-Positive Tumors Need Specific Predictors. Breast Cancer Res Treat (2012) 132(2):601–7. doi: 10.1007/s10549-011-1897-0 22160638

[100] Uhlig J Uhlig A Biggemann L Fischer U Lotz J Wienbeck S . Diagnostic Accuracy of Cone-Beam Breast Computed Tomography: A Systematic Review and Diagnostic MetaAnalysis. Eur Radiol (2019) 29(3):1194–202. doi: 10.1007/s00330-018-5711-9 30255249

[101] Deeks JJ . Systematic Reviews of Evaluations of Diagnostic and Screening Tests. In: Egger M Smith GD Altman DG , editors. Systematic Reviews in Health Care: Meta-Analysis in Context. London, UK: BMJ Publishing Group (2001). p. 248e82. 10.1136/bmj.323.7305.157 PMC1120791 11463691

[102] Jaeschke R Guyatt G Lijmer J . Diagnostic Tests. In: Guyatt G Rennie D , editors. Users’ Guides to the Medical Literature: A Manual for Evidence-Based Clinical Practice. Chicago, IL: AMA Press (2002). p. 121e40.

[103] Ferrari A Vincent-Salomon A Pivot X Sertier A-S Thomas E Tonon L . A Whole-Genome Sequence and Transcriptome Perspective on HER2-Positive Breast Cancers. Nat Commun (2016) 7:12222. doi: 10.1038/ncomms12222 27406316 PMC4947184

[104] Prat A Carey LA Adamo B Vidal M Tabernero J Cortés J . Molecular Features and Survival Outcomes of the Intrinsic Subtypes Within HER2-Positive Breast Cancer. J Natl Cancer Inst (2014) 106(8):dju152. doi: 10.1093/jnci/dju152 25139534 PMC4151853

[105] Cejalvo JM Pascual T Fernández-Martínez A Adamo B Chic N Vidal M . 1727pdistribution of the ´PAM50 Breast Cancer Subtypes Within Each Pathology-Based Group: A Combined Analysis of 15,339 Patients Across 29 Studies. Ann Oncol (2017) 28(suppl_5):V603. doi: 10.1093/annonc/mdx391.026

[106] Bender LM Nahta R . Her2 Cross Talk and Therapeutic Resistance in Breast Cancer. Front Biosc: J Virtual Library (2008) 13:3906. doi: 10.2741/2978 PMC2746389 18508484

