# Supplementary material for: Comparing Biomarkers for Predicting Pathological Responses to Neoadjuvant Therapy in HER2-Positive Breast Cancer: A Systematic Review and Meta-Analysis
Source: Front Oncol. 2021 Oct 28;11:731148. doi: 10.3389/fonc.2021.731148 (PMC8581664; doi:10.3389/fonc.2021.731148)
Supplement: Supplementary Table 1 — Systematic review search strategy. [file Table_1.doc]

| **Table S1. Systematic review search strategy.** | |
| --- | --- |
| **Medline** | |
| Breast cancer |  |
| MeSH terms | breast neoplasms or carcinoma or inflammatory breast neoplasms or unilateral breast neoplasms or malignant neoplasm of breast or malignant tumor of breast or human mammary carcinomas |
| Keywords | breast cancer *or breast neoplasm or breast tumour or breast adenocarcinoma or breast carcinogenesis or breast carcinoma or breast sarcoma |
| Neoadjuvant therapy |  |
| MeSH terms | neoadjuvant chemotherapy or NACT or NCT |
| Keywords | neoadjuvant chemotherapy *or NACT or NCT |
| HER2-positive |  |
| MeSH terms | HER 2-positive or Her 2-positive or ERBB 2 positive or ErbB 2 gene expression |
| Keywords | HER 2-positive *or Her 2-positive or ERBB 2 positive or ErbB 2 gene expression |
| Pathological complete response |  |
| MeSH terms | pathological complete response or pathological response or pathological remission or pCR |
| Keywords | pathological complete response *or pathological response or pathological remission or pCR |

| **Embase** | |
| --- | --- |
| Breast cancer |  |
| MeSH terms | breast cancer or breast neoplasms or carcinoma or inflammatory breast neoplasms or unilateral breast neoplasms or carcinoma or mammary cancer |
| Keywords | breast cancer *or breast neoplasm or breast tumor or breast adenocarcinoma or breast carcinogenesis or breast sarcoma or carcinoma |
| Neoadjuvant therapy |  |
| MeSH terms | neoadjuvant chemotherapy or NACT or NCT or preoperative chemotherapy or neoadjuvant treatments |
| Keywords | neoadjuvant chemotherapy*or NACT or NCT or preoperative chemotherapy or preoperative chemotherapy |
| HER2-positive |  |
| MeSH terms | HER2-positive or ERBB2 positive or ErbB 2 gene positive |
| Keywords | HER2-positive *or ERBB2 positive or ErbB 2 gene positive |
| Pathological complete response |  |
| MeSH terms | pathological complete response or pathological response or pathological remission or pCR |
| Keywords | pathological complete response *or pathological response or pathological remission or pCR |

| **Pubmed** | |
| --- | --- |
| Breast cancer |  |
| MeSH terms | breast neoplasms or carcinoma or inflammatory breast neoplasms or unilateral breast neoplasms |
| Keywords | breast cancer*or breast neoplasm or breast tumour or breast adenocarcinoma or breast carcinogenesis or breast carcinoma or breast sarcoma |
| Neoadjuvant therapy |  |
| MeSH terms | neoadjuvant chemotherapy or NACT or NCT or neoadjuvant treatments |
| Keywords | neoadjuvant chemotherapy* or NACT or NCT or neoadjuvant treatments |
| HER2-positive |  |
| MeSH terms | HER2-positive or ERBB2 positive |
| Keywords | HER2-positive *or ERBB2 positive |
| pathological complete response |  |
| MeSH terms | pathological complete response or pathological response or pathological remission or pCR |
| Keywords | pathological complete response *or pathological response or pathological remission or pCR |
