# Supplementary material for: Comparing Biomarkers for Predicting Pathological Responses to Neoadjuvant Therapy in HER2-Positive Breast Cancer: A Systematic Review and Meta-Analysis
Source: Front Oncol. 2021 Oct 28;11:731148. doi: 10.3389/fonc.2021.731148 (PMC8581664; doi:10.3389/fonc.2021.731148)
Supplement: Supplementary Table 2 — Characteristics of the studies included. [file Table_2.doc]

**Table S2. Summary of research characteristics.**

| **Index** | **Author** | **Year** | **Study design** | **TNM** | **Group** | **T** | **P** | **L** | **T-DM1** | **Chemotherapy drugs** | **N** | **pCR** | **Non-pCR** | **pCR** | **Non-pCR** | **pCR definition** |
| --- | --- | --- | --- | --- | --- | --- | --- | --- | --- | --- | --- | --- | --- | --- | --- | --- |
|  |  |  |  |  |  |  |  |  |  |  |  | **LPBC** | | **Non-LPBC** | |  |
| **TIL** | Denkert et al. | 2014 | Prospective | NA | PM | √ |  | √ |  | 18 weeks with paclitaxel 80 mg/m^2^ once every week and nonpegylated liposomal doxorubicin 20 mg/m^2^ once everyweek | 134 | 13 | 13 | 36 | 72 | ypT0 ypN0 |
|  | . |  |  |  | PMCb | √ |  | √ |  | 18 weeks with paclitaxel 80 mg/m^2^ once every week and nonpegylated liposomal doxorubicin 20 mg/m^2^ once everyweek plus carboplatin | 132 | 21 | 6 | 23 | 82 | ypT0 ypN0 |
| **TIL** | Salgado et al. | 2015 | Prospective | NA | T | √ |  |  |  | Weekly paclitaxel for 12 weeks | 131 | 26 | 80 | 8 | 17 | ypT0/is ypN0 |
|  |  |  |  |  | L |  |  | √ |  | Weekly paclitaxel for 12 weeks | 130 | 19 | 77 | 7 | 27 | ypT0/is ypN0 |
|  |  |  |  |  | T+L | √ |  | √ |  | Weekly paclitaxel for 12 weeks | 96 | 45 | 50 | 15 | 16 | ypT0/is ypN0 |
| **TIL** | Dieci et al. | 2016 | Prospective | II-IIIA | Arm A | √ |  |  |  | Weekly paclitaxel followed by FEC (fluorouracil, epirubicin and cyclophosphamide) | 32 | 2 | 3 | 7 | 20 | ypT0/is ypN0 |
|  |  |  |  |  | Arm B |  |  | √ |  | Weekly paclitaxel followed by FEC (fluorouracil, epirubicin and cyclophosphamide) | 34 | 4 | 2 | 4 | 24 | ypT0/is ypN0 |
|  |  |  |  |  | Arm C | √ |  | √ |  | Weekly paclitaxel followed by FEC (fluorouracil, epirubicin and cyclophosphamide) | 39 | 5 | 1 | 11 | 22 | ypT0/is ypN0 |
| **TIL** | Heppner et al. | 2016 | Prospective | NA |  |  |  | √ |  | Patients received four cycles of epirubicin/cyclophosphamide (EC) followed by four cycles of  docetaxel with or without capecitabine. | 219 | 35 | 25 | 64 | 95 | ypT0/is ypN0 |
| **TIL** | Angelis et al. | 2019 | Prospective | II-III |  | √ |  | √ |  | ER and/or PR positive patients received endocrine therapy. | 59 | 6 | 6 | 9 | 38 | NA |
|  |  |  |  |  |  |  |  |  |  |  | **N** | **Ki67 High** | | **Ki67 Low / Medium** | |  |
| **Ki-67** | Esserman et al. | 2012 | Prospective | I-III |  | √ |  |  |  | Patients could either undergo surgical excision or receive a taxane before surgery. | 39 | 5 | 12 | 8 | 14 | NA |
| **Ki-67** | Saracchini et al. | 2013 | Prospective | IIA-IIIC |  | √ |  |  |  | Liposome-encapsulated doxorubicin (60 mg/mq iv) plus cyclophosphamide (600 mg/mq iv) every 3 weeks for 4 cycles followed by docetaxel (35 mg/mq iv) | 38 | 18 | 12 | 1 | 7 | ypT0/is ypN0 |
| **Ki-67** | Kurozumi et al. | 2015 | Prospective | I-III |  | √ |  |  |  | Patients received 12 cycles of paclitaxel (80 mg/m^2^ ) every week or 4 cycles of docetaxel (75 mg/m^2^ ) every 3 weeks followed by 4 cycles of FEC-75 (5-fluorouracil, 500 mg/m2 , epirubicin, 75 mg/ m^2^ ; and cyclophosphamide, 500 mg/m^2^ ) every 3 weeks. | 129 | 67 | 26 | 17 | 19 | ypT0/is |
| **Ki-67** | Huang et al. | 2013 | Retrospective | II-III |  | √ |  |  |  | All patients were treated with CEF (cyclophosphamide 600 mg/m^2^ , epirubicin 80 mg/m^2^ and fluorouracil 500 mg/m^2^, q3w) or NE (vinorelbine 25 mg/m^2^ on days 1 and 8 and epirubicin 60 mg/m^2^  on day 1, q3w). | 113 | 12 | 58 | 2 | 41 | ypT0/is ypN0 |
| **Ki-67** | Zhang et al. | 2012 | Retrospective | II-III |  | √ |  |  |  | TCH docetaxel, carboplatinum, and trastuzumab, DH docetaxel and trastuzumab, TH paclitaxel and trastuzumab | 102 | 28 | 21 | 17 | 36 | ypT0 ypN0 |
| **Ki-67** | ALBA et al. | 2016 | Prospective | NA | ER+/HER2+ | √ |  | √ |  | Patients were treated with anthracycline and taxane-based neoadjuvant chemotherapy. | 62 | 3 | 9 | 12 | 39 | ypT0/is ypN0 |
|  | . |  |  |  | ER-/HER2+ | √ |  | √ |  | Patients were treated with anthracycline and taxane-based neoadjuvant chemotherapy. | 51 | 7 | 4 | 18 | 22 | ypT0/is ypN0 |
| **Ki-67** | Bria et al. | 2015 | Prospective | II-III |  | √ |  |  |  | Patients were treated with docetaxel or Taxotere or Sanofi-Aventis (100 mg/m2 every 3 weeks) | 24 | 0 | 5 | 4 | 15 | ypT0/is ypN0 |
| **Ki-67** | Kim et al. | 2014 | Prospective | II-III | ER+/HER2+ |  |  |  |  | Six cycles of AT (50 mg/m2 doxorubicin and 75 mg/m2 docetaxel every 3 weeks), four cycles of CEF (500 mg/m2 cyclophosphamide, 100 mg/m2 epirubicin, and 500 mg/m2 fluorouracil on days 1  and 8 every 3 weeks), and four cycles of CAF (500 mg/m2 cyclophosphamide, 50 mg/m2 doxorubicin, and 500 mg/m2 fluorouracil every 3 weeks). | 15 | 2 | 8 | 0 | 5 | ypT0/is ypN0 |
|  |  |  |  |  | ER-/HER2+ |  |  |  |  | Six cycles of AT (50 mg/m2 doxorubicin and 75 mg/m2 docetaxel every 3 weeks), four cycles of CEF (500 mg/m2 cyclophosphamide, 100 mg/m2 epirubicin, and 500 mg/m2 fluorouracil on days 1  and 8 every 3 weeks), and four cycles of CAF (500 mg/m2 cyclophosphamide, 50 mg/m2 doxorubicin, and 500 mg/m^2^ fluorouracil every 3 weeks). | 20 | 5 | 7 | 1 | 7 | ypT0/is ypN0 |
| **Ki-67** | Ding et al. | 2017 | Prospective | NA |  | √ |  |  |  | Paclitaxel, Carboplatin and Trastuzumab. | 88 | 19 | 20 | 11 | 38 | ypT0/is ypN0 |
| **Ki-67** | Harbeck et al. | 2017 | Prospective |  | T-DM1 |  |  |  | √ | / | 97 | 24 | 37 | 9 | 27 | ypT0/is/ypN0 |
|  |  |  | Prospective |  | T-DM1+ET |  |  |  | √ | Endocrine therapy. | 101 | 36 | 40 | 6 | 19 | ypT0/is/ypN0 |
|  |  |  | Prospective |  | H+ET | √ |  |  |  | Endocrine therapy. | 102 | 11 | 51 | 5 | 35 | ypT0/is/ypN0 |
|  |  |  |  |  |  |  |  |  |  |  | **N** | **HR negative** | | **HR positive** | |  |
| **HR** | Carey et al. | 2016 | Prospective | II-III | THL | √ |  | √ |  | Patients received paclitaxel intravenously at 80 mg/m2 once per week for 16 weeks. | 116 | 37 | 10 | 28 | 41 | ypT0/is |
|  |  |  |  |  | TH | √ |  |  |  | Patients received paclitaxel intravenously at 80 mg/m2 once per week for 16 weeks. | 117 | 26 | 22 | 28 | 41 | ypT0/is |
|  |  |  |  |  | TL |  |  | √ |  | Patients received paclitaxel intravenously at 80 mg/m2 once per week for 16 weeks. | 64 | 10 | 17 | 11 | 26 | ypT0/is |
| **HR** | Bonnefoi et al. | 2015 | Prospective | II-III | L |  |  | √ |  | Received six cycles of chemotherapy (three cycles of docetaxel followed by three cycles of fluorouracil, epirubicin, cyclophosphamide). | 22 | 2 | 6 | 8 | 6 | ypT0/is |
|  |  |  | Prospective |  | T | √ |  |  |  | Received six cycles of chemotherapy (three cycles of docetaxel followed by three cycles of fluorouracil, epirubicin, cyclophosphamide). | 52 | 13 | 12 | 14 | 13 | ypT0/is |
|  |  |  | Prospective |  | T+L | √ |  | √ |  | Received six cycles of chemotherapy (three cycles of docetaxel followed by three cycles of fluorouracil, epirubicin, cyclophosphamide). | 48 | 17 | 8 | 12 | 11 | ypT0/is |
| **HR** | Untch et al. | 2010 | Prospective | NA |  | √ |  |  |  | Four cycles of epirubicin/cyclophosphamide followed by four cycles of docetaxel with or without capecitabine (EC-T[X]) | 445 | 80 | 104 | 61 | 200 | ypT0 |
| **HR** | Alba et al. | 2014 | Prospective | I-III |  | √ |  | √ |  | Receive epirubicin (E) plus cyclophosphamide (C) 4 cycles followed by docetaxel (D) | 102 | 29 | 14 | 20 | 39 | ypT0/is |
| **HR** | Holmes et al. | 2013 | Prospective | I-III | T | √ |  |  |  | FEC： 5-fluorouracil 500 mg/m^2^,epirubicin 75 mg/m^2^ , cyclophosphamide 500 mg/m^2^. | 33 | 12 | 6 | 6 | 9 | ypT0/is ypN0 |
|  |  |  |  |  | L |  |  | √ |  | FEC： 5-fluorouracil 500 mg/m^2^,epirubicin 75 mg/m^2^ , cyclophosphamide 500 mg/m^2^. | 34 | 10 | 10 | 3 | 11 | ypT0/is ypN0 |
|  |  |  |  |  | T+L | √ |  | √ |  | FEC： 5-fluorouracil 500 mg/m^2^,epirubicin 75 mg/m^2^ , cyclophosphamide 500 mg/m^2^. | 33 | 6 | 7 | 12 | 8 | ypT0/is ypN0 |
| **HR** | Baselga et al. | 2012 | Prospective | NA | L |  |  | √ |  | Weekly paclitaxel (80 mg/m2 ) was then added to the regimen for a further 12 weeks. | 154 | 25 | 49 | 13 | 67 | ypT0/is ypN0 |
|  |  |  |  |  | T | √ |  |  |  | Weekly paclitaxel (80 mg/m2 ) was then added to the regimen for a further 12 weeks. | 149 | 27 | 47 | 17 | 58 | ypT0/is ypN0 |
|  |  |  |  |  | T+L | √ |  | √ |  | Weekly paclitaxel (80 mg/m2 ) was then added to the regimen for a further 12 weeks. | 152 | 46 | 29 | 32 | 45 | ypT0/is ypN0 |
| **HR** | Gianni et al. | 2012 | Prospective | NA | T+D | √ |  |  |  | Docetaxel (75 mg/m², escalating, if tolerated, to 100 mg/m² every 3 weeks). | 107 | 21 | 36 | 10 | 40 | ypT0/is |
|  |  |  |  |  | P+T+D | √ | √ |  |  | Docetaxel (75 mg/m², escalating, if tolerated, to 100 mg/m² every 3 weeks). | 107 | 36 | 21 | 13 | 37 | ypT0/is |
|  |  |  |  |  | P+T | √ | √ |  |  | Docetaxel (75 mg/m², escalating, if tolerated, to 100 mg/m² every 3 weeks). | 106 | 15 | 40 | 3 | 48 | ypT0/is |
|  |  |  |  |  | P+D |  | √ |  |  | Docetaxel (75 mg/m², escalating, if tolerated, to 100 mg/m² every 3 weeks). | 96 | 15 | 35 | 8 | 38 | ypT0/is |
| **HR** | Robidoux et al. | 2013 | Prospective | NA | T | √ |  |  |  | Four cycles of standard doxorubicin 60 mg/m² and cyclophosphamide 600 mg/m² intravenously on day 1 every 3 weeks followed by four cycles of weekly paclitaxel (80 mg/m²) intravenously on days 1, 8, and 15, every 4 weeks. | 177 | 36 | 19 | 57 | 65 | ypT0/is |
|  |  |  |  |  | L |  |  | √ |  | Four cycles of standard doxorubicin 60 mg/m² and cyclophosphamide 600 mg/m² intravenously on day 1 every 3 weeks followed by four cycles of weekly paclitaxel (80 mg/m²) intravenously on days 1, 8, and 15, every 4 weeks. | 171 | 43 | 28 | 48 | 52 | ypT0/is |
|  |  |  |  |  | T+L | √ |  | √ |  | Four cycles of standard doxorubicin 60 mg/m² and cyclophosphamide 600 mg/m² intravenously on day 1 every 3 weeks followed by four cycles of weekly paclitaxel (80 mg/m²) intravenously on days 1, 8, and 15, every 4 weeks. | 171 | 46 | 17 | 60 | 48 | ypT0/is |
| **HR** | Schneeweiss et al. | 2013 | Prospective | NA |  | √ |  | √ |  | Receive six neoadjuvant cycles q3w ( 5-fluorouracil, epirubicin, cyclophosphamide×3→docetaxel [T] ×3). | 225 | 84 | 27 | 55 | 59 | ypT0/is |
| **HR** | Pierga et al. | 2010 | Prospective | II-III |  | √ |  |  |  | Eight sequential three weekly cycles of EC-D [epirubicin (75 mg/m^2^)–cyclophosphamide (750 mg/m^2^ ) for four cycles followed by docetaxel (100 mg/m^2^ ) for four cycles | 62 | 9 | 19 | 7 | 27 | ypT0/is ypN0 |
| **HR** | Patel et al. | 2019 | Prospective | II-III | S | √ | √ |  |  | Receive paclitaxel 80 mg/m2 weekly. | 16 | 8 | 0 | 2 | 6 | ypT0/is ypN0 |
|  |  |  | Prospective |  | E |  |  | √ | √ | Receive paclitaxel 80 mg/m2 weekly. | 14 | 6 | 0 | 6 | 2 | ypT0/is ypN0 |
| **HR** | I-SPY2 | 2020 | Prospective | II-III |  | √ | √ |  |  | 12 weekly cycles of paclitaxel (80 mg/m^2^) followed by 4 cycles of doxorubicin (60 mg/m^2^) and cyclophosphamide (600 mg/m^2^) | 263 | 61 | 29 | 69 | 104 | NA |
|  |  |  |  |  |  |  |  |  |  |  | **N** | **PIK3CA wt** | | **PIK3CA mutation** | |  |
| **PIK3CA** | Loibl et al. | 2016 | Prospective | NA | T | √ |  |  |  | Taxane-based chemotherapy. | 315 | 68 | 183 | 13 | 51 | ypT0 ypN0 |
|  |  |  |  |  | L |  |  | √ |  | Taxane-based chemotherapy. | 251 | 32 | 157 | 7 | 55 | ypT0 ypN0 |
|  |  |  |  |  | T+L | √ |  | √ |  | Taxane-based chemotherapy. | 399 | 123 | 192 | 14 | 70 | ypT0 ypN0 |
| **PIK3CA** | Bianchini et al. | 2017 | Prospective | NA | T+D | √ |  |  |  | Anthracycline-taxane chemotherapy. | 70 | 15 | 28 | 6 | 21 | ypT0/is |
|  |  |  |  |  | P+T+D | √ |  |  |  | Anthracycline-taxane chemotherapy. | 69 | 25 | 24 | 7 | 13 | ypT0/is |
|  |  |  |  |  | P+T | √ | √ |  |  | Anthracycline-taxane chemotherapy. | 71 | 8 | 42 | 2 | 19 | ypT0/is |
|  |  |  |  |  | P+D |  | √ |  |  | Anthracycline-taxane chemotherapy. | 63 | 14 | 29 | 4 | 16 | ypT0/is |
| **PIK3CA** | Dave et al. | 2011 | Prospective |  | L |  |  | √ |  | Docetaxel every 3 weeks for 12 weeks. | 31 | 16 | 10 | 3 | 2 | ypT0 |
|  |  |  |  |  | H | √ |  |  |  | Docetaxel every 3 weeks for 12 weeks. | 31 | 8 | 13 | 2 | 8 | ypT0 |
| **PIK3CA** | Harbeck et al. | 2016 | Prospective | NA |  | √ |  |  | √ | NA | 373 | 96 | 175 | 18 | 84 | ypT0/is ypN0 |
| **PIK3CA** | Carey et al. | 2016 | Prospective | II-III |  | √ |  | √ |  | Patients received paclitaxel intravenously at 80 mg/m2 once per week for 16 weeks. | 181 | 68 | 77 | 14 | 22 | ypT0/is |
| **PIK3CA** | Sueta et al. | 2014 | Prospective | NA |  | √ |  |  |  | NA | 43 | 24 | 12 | 2 | 5 | ypT0/is ypN0 |
| **PIK3CA** | Loibl et al. | 2017 | Prospective | NA | BH | √ |  |  |  | NA | 25 | 7 | 14 | 1 | 3 | ypT0/is |
|  |  |  |  |  | H | √ |  |  |  | NA | 25 | 9 | 12 | 1 | 3 | ypT0/is |
| **PIK3CA** | Rimawi et al. | 2017 | Prospective | II-III |  | √ |  | √ |  | Patients with ER+ and/or PR positive were treated with letrozole 2.5 mg orally once per day (combined with LHRH agonist of choice in premenopausal women). | 46 | 9 | 23 | 1 | 13 | ypT0/is |
| **PIK3CA** | Guarneri et al. | 2014 | Prospective | NA | L |  |  | √ |  | NA | 159 | 26 | 96 | 6 | 31 | NA |
|  |  |  |  |  | T | √ |  |  |  | NA | 142 | 31 | 84 | 6 | 21 | NA |
|  |  |  |  |  | L+T | √ |  | √ |  | NA | 401 | 144 | 173 | 18 | 66 | NA |
| **PIK3CA** | Schneeweiss et al. | 2014 | Prospective | NA |  | √ | √ |  |  | Arm A：5-fluorouracil/epirubicin/cyclophosphamide (FEC) for cycles one to three and docetaxel for cycles four to six. Arm B：FEC for cycles one to three. Arm C：Docetaxel and carboplatin. | 165 | 81 | 45 | 19 | 20 | ypT0 ypN0 |
| **PIK3CA** | Majewski et al. | 2015 | Prospective | NA | L |  |  | √ |  | Patients were treated with paclitaxel for 12 weeks. | 120 | 19 | 74 | 4 | 23 | ypT0/is ypN0 |
|  |  |  |  |  | T | √ |  |  |  | Patients were treated with paclitaxel for 12 weeks. | 108 | 25 | 63 | 4 | 16 | ypT0/is ypN0 |
|  |  |  |  |  | L+T | √ |  | √ |  | Patients were treated with paclitaxel for 12 weeks. | 114 | 48 | 38 | 8 | 20 | ypT0/is ypN0 |
|  |  |  |  |  |  |  |  |  |  |  | **N** | **HER2-enriched** | | **non-HER2-enriched** | |  |
| **HER-2 E** | Prat et al. | 2019 | Prospective | II-III |  |  |  | √ |  | Women with ER-positive tumors received letrozole (plus a luteinizing hormone–releasing hormone [LHRH] agonist if premenopausal) | 114 | 20 | 53 | 4 | 37 | ypT0/is |
| **HER-2 E** | Prat et al. | 2018 | Prospective | II-III |  |  |  | √ |  | Women with ER-positive tumors received letrozole (plus a luteinizing hormone–releasing hormone [LHRH] agonist if premenopausal) | 85 | 14 | 37 | 3 | 31 | ypT0/is |
| **HER-2 E** | Antonio et al. | 2017 | Prospective | I-IIIA |  |  |  | √ |  | Hormone receptor-positive patients were additionally given letrozole (2.5 mg per day orally; if menopausal) or tamoxifen (20 mg per day orally; if premenopausal). | 151 | 41 | 60 | 5 | 45 | NA |
| **HER-2 E** | V Guarneri et al. | 2019 | Prospective | II-IIIA |  |  | √ |  |  | NA | 40 | 5 | 6 | 4 | 25 | ypT0/is ypN0 |
| **HER-2 E** | Joaquín et al. | 2019 | Prospective | II-IIIB |  |  | √ |  |  | Paclitaxel, and a non-pegylated liposomal doxorubicin every three weeks for six cycles. | 55 | 24 | 6 | 13 | 15 | ypT0/isypN0 |
| **HER-2 E** | Debora et al. | 2014 | Prospective | NA |  |  |  | √ |  | Weekly paclitaxel (80 mg/m²). | 244 | 57 | 53 | 31 | 113 | ypT0/isypN0 |
| **HER-2 E** | Valentina et al. | 2016 | Prospective | II-IIIA |  |  |  | √ |  | Weekly paclitaxel (80 mg/m2) for 12 weeks followed by fluorouracil, epirubicin, and cyclophosphamide for four courses every 3 weeks. | 69 | 11 | 11 | 8 | 39 | ypT0/is ypN0 |
| **HER-2 E** | S. M. Swain et al. | 2018 | Prospective | NA | Cohort A |  |  |  |  | Patients received four cycles of dose-dense doxorubicin and cyclophosphamide, then 12 doses of standard paclitaxel plus. | 199 | 60 | 20 | 63 | 56 | ypT0/is ypN0 |
|  |  |  |  |  | Cohort B |  |  |  |  | Patients received four standard fluorouracil/ epirubicin/ cyclophosphamide cycles, then four docetaxel cycles. | 201 | 70 | 25 | 52 | 54 | ypT0/is ypN0 |
| **HER-2 E** | Cheang et al. | 2009 | Prospective | II-III |  | √ |  |  |  | All patients received four cycles of capecitabine (825 mg/m2 orally twice daily on days 1–14) plus docetaxel 75 mg/m2 intravenously (IV) on day 1, every 3 weeks. | 24 | 7 | 5 | 1 | 11 | ypT0/is |
| **HER-2 E** | Swain et al. | 2019 | Prospective | NA | L |  |  | √ |  | NA | 95 | 34 | 35 | 9 | 17 | ypT0/Tis ypN0 |
|  |  |  |  |  | H | √ |  |  |  | NA | 94 | 46 | 25 | 4 | 19 | ypT0/Tis ypN0 |
|  |  |  |  |  | HL | √ |  | √ |  | NA | 82 | 40 | 17 | 6 | 19 | ypT0/Tis ypN0 |
| **HER-2 E** | Prat et al. | 2014 | Prospective | NA |  | √ |  |  |  | Receive neoadjuvant doxorubicin/paclitaxel (AT) followed by cyclophosphamide methotrexate/ fluorouracil ( CMF) . | 63 | 18 | 16 | 10 | 19 | ypT0/Tis ypN0 |
| **HER-2 E** | Prat et al. | 2015 | Prospective | NA |  | √ |  |  |  | All patients received sequential anthracycline and taxane/ exabepilone-based neoadjuvant regimens. | 50 | 12 | 14 | 4 | 20 | ypT0/is |
| **HER-2 E** | Prat et al. | 2017 | Prospective | II-IIIC | T-DM1+P |  | √ |  | √ | Docetaxel + carboplatin. | 183 | 56 | 34 | 25 | 68 | ypT0/is, ypN0 |
|  |  |  |  |  | HP | √ | √ |  |  | / | 171 | 75 | 29 | 22 | 45 | ypT0/is, ypN0 |
| **HER-2 E** | Nakatsukasa et al. | 2016 | Prospective | I-III |  | √ |  |  |  | Docetaxel (75 mg/m2), cyclophosphamide (600 mg/m2). | 41 | 10 | 11 | 8 | 12 | ypT0/TisypN0 |
| **HER-2 E** | Carey et al. | 2015 | Prospective | II-III | TH | √ |  |  |  | Patients received paclitaxel intravenously at 80 mg/m2 once per week for 16 weeks. | 104 | 17 | 7 | 30 | 50 | ypT0/is |
|  |  |  |  |  | THL | √ |  | √ |  | Patients received paclitaxel intravenously at 80 mg/m2 once per week for 16 weeks. | 103 | 28 | 7 | 27 | 41 | ypT0/is |
|  |  |  |  |  | TL |  |  | √ |  | Patients received paclitaxel intravenously at 80 mg/m2 once per week for 16 weeks. | 58 | 12 | 11 | 7 | 28 | ypT0/is |
| **HER-2 E** | Lesurf et al. | 2017 | Prospective | NA |  | √ |  |  |  | NA | 42 | 11 | 3 | 11 | 17 | NA |
| HR：Hormone receptor; PIK3CA：phosphatase phosphoinositol-3 (PI3) kinase; TIL：Tumor-infiltrating lymphocytes; LPBC:lymphocyte-predominant breast cancer；NA：not availble; T：Trastuzumab；P：Patuzumab；L：Lapatinib；ET：endocrine therapy；D：Docetaxel；HER-2 E：HER-2 Enriched. | | | | | | | | | | | | | | | | |
