# Supplementary material for: Comparing Biomarkers for Predicting Pathological Responses to Neoadjuvant Therapy in HER2-Positive Breast Cancer: A Systematic Review and Meta-Analysis
Source: Front Oncol. 2021 Oct 28;11:731148. doi: 10.3389/fonc.2021.731148 (PMC8581664; doi:10.3389/fonc.2021.731148)
Supplement: Supplementary Figure 2 — Forest plots of sensitivity and specificity. 2A. Sensitivity. 2B. Specificity. [file DataSheet_2.doc]

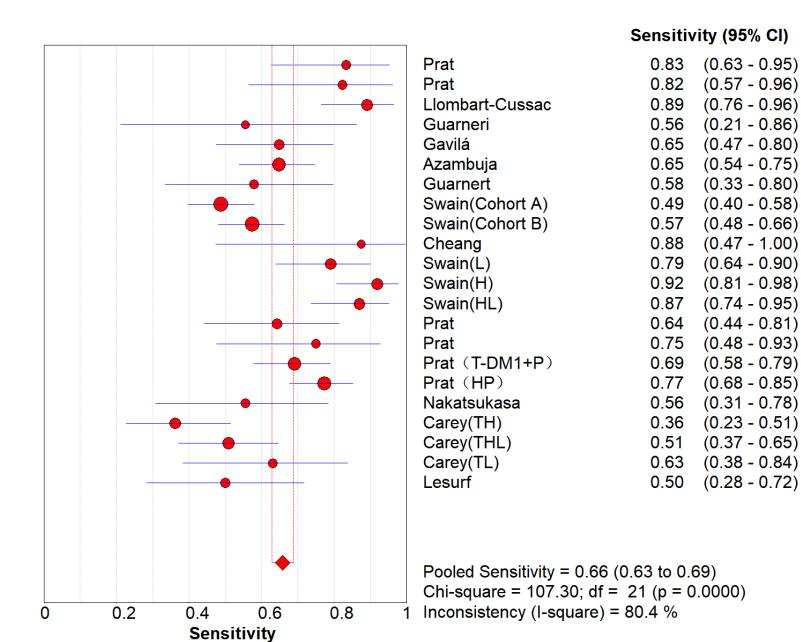


**A**


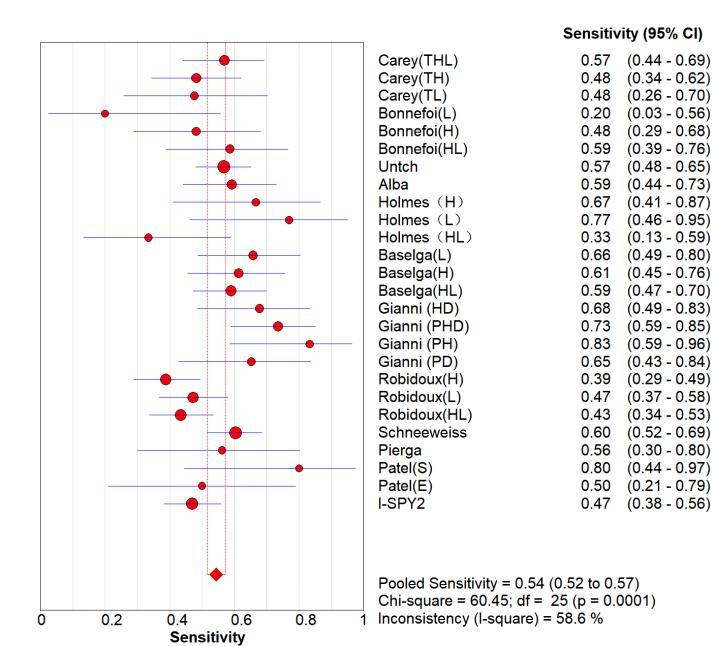


**B**


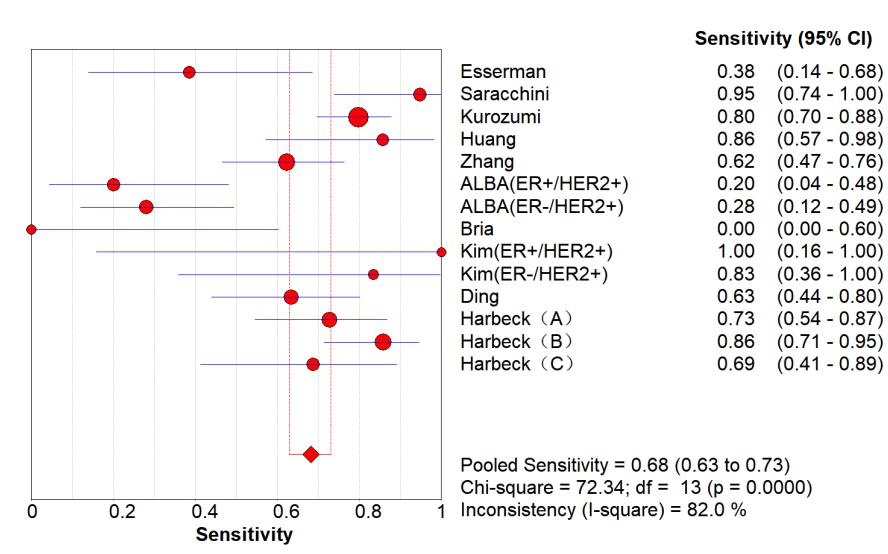


**C**


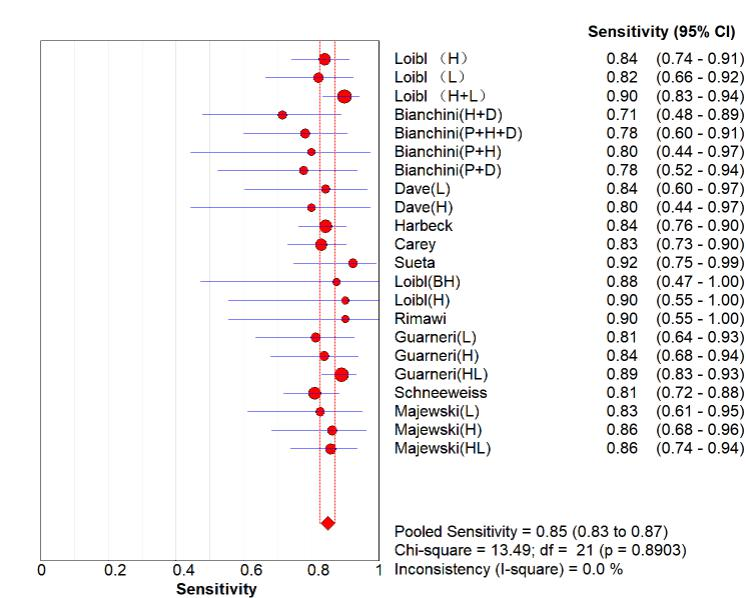


**D**


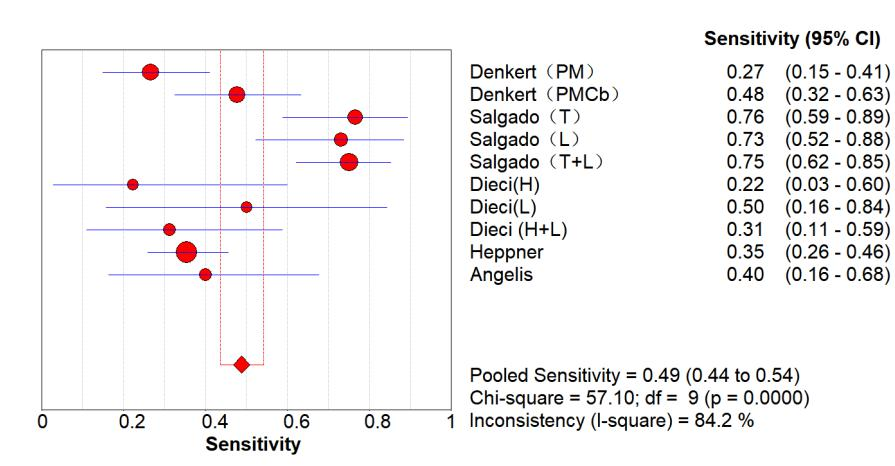


**E**

A：Her-2 enriched B：Hormone receptor C：Ki-67 D：phosphatase phosphoinositol-3 (PI3) kinase E：Tumor-infiltrating lymphocytes

**Figure S2A**


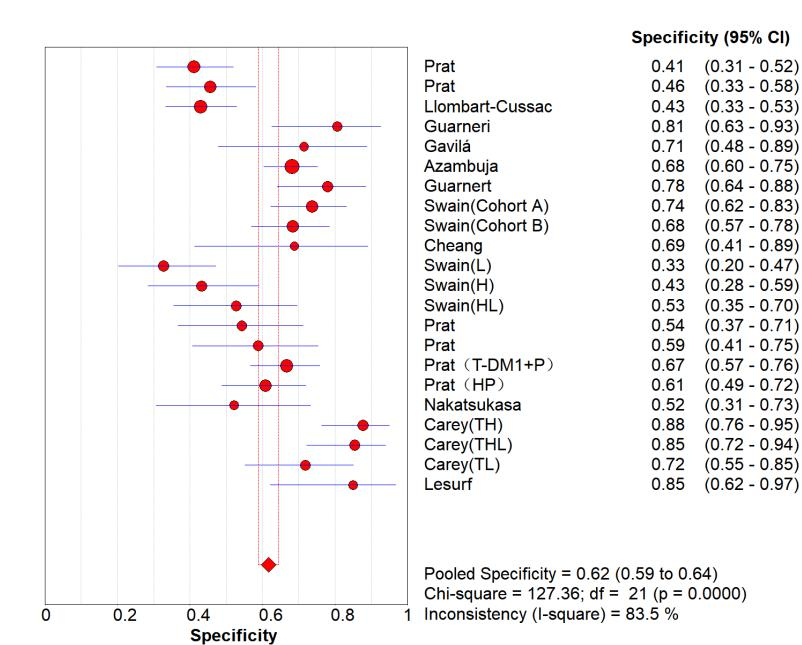


**A**


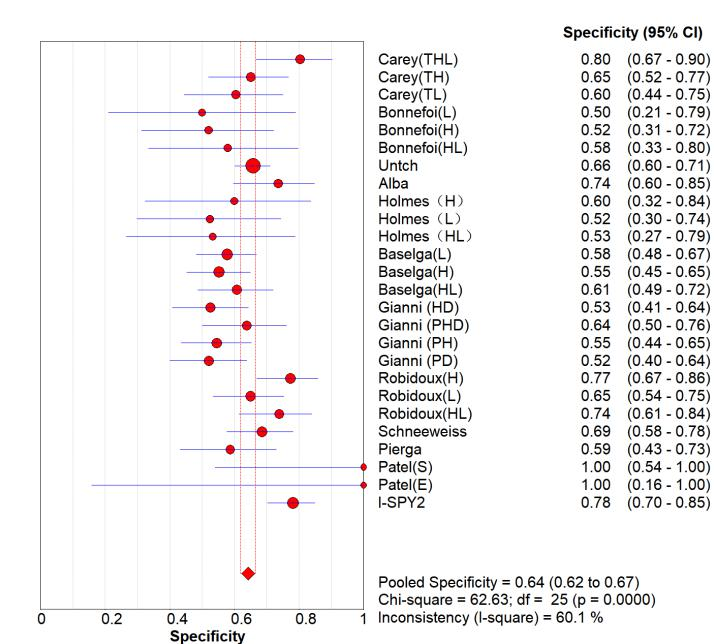


**B**


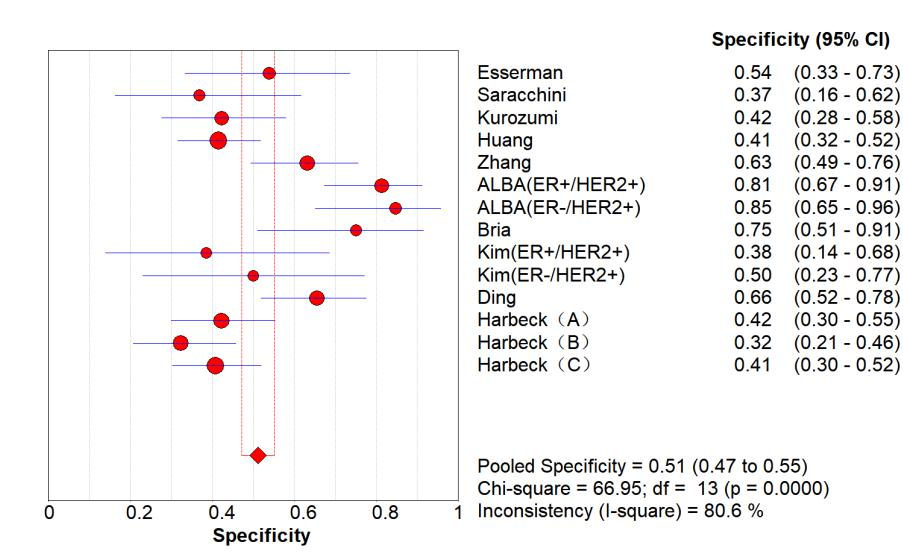


**C**


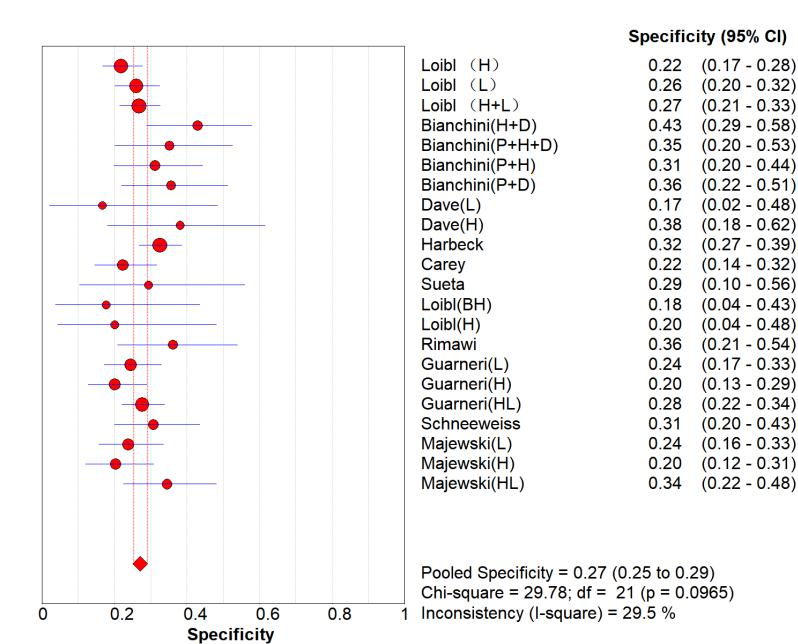


**D**


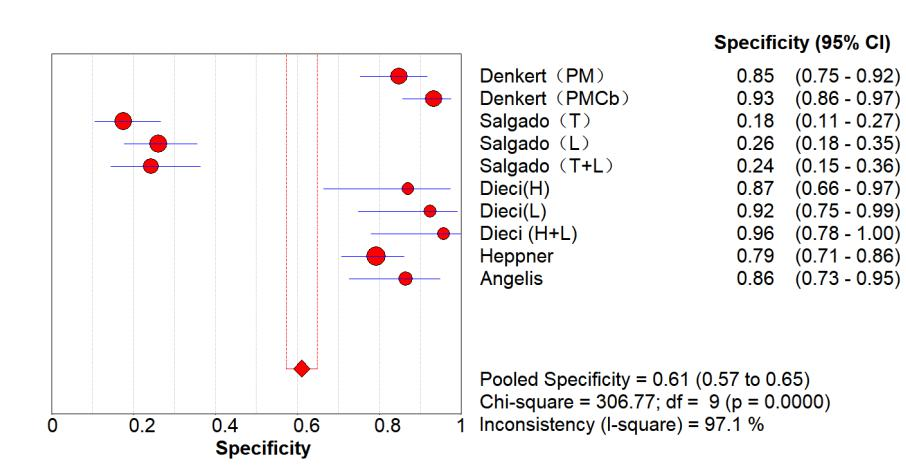


**E**

A：Her-2 enriched B：Hormone receptor C：Ki-67 D：phosphatase phosphoinositol-3 (PI3) kinase E：Tumor-infiltrating lymphocytes

**Figure S2B**
